# Dimeric Her2-specific affibody mediated cisplatin-loaded nanoparticles for tumor enhanced chemo-radiotherapy

**DOI:** 10.1186/s12951-021-00885-6

**Published:** 2021-05-13

**Authors:** Haijun Wang, Dianlong Jia, Dandan Yuan, Xiaolei Yin, Fengjiao Yuan, Feifei Wang, Wenna Shi, Hui Li, Li-Min Zhu, Qing Fan

**Affiliations:** 1 Department of Pharmacy, Shandong Cancer Hospital and Institute, Shandong First Medical University and Shandong Academy of Medical Sciences, Jinan, 250117 China; 2 College of Chemistry, Chemical Engineering and Biotechnology, Donghua University, Shanghai, 201620 China; 3 Laboratory of Drug Discovery and Design, School of Pharmacy, Liaocheng University, Liaocheng, 252000 China; 4 School of Life Sciences, Shandong First Medical University and Shandong Academy of Medical Sciences, Taian, 271016 China; 5 Department of Digestive Oncology, Shandong Cancer Hospital and Institute, Shandong First Medical University and Shandong Academy of Medical Sciences, Jinan, 250117 China; 6 Joint Laboratory for Translational Medicine Research, Liaocheng People’s Hospital, Liaocheng, 252000 China

**Keywords:** Mesoporous polydopamine, MnO_2_, Dimeric Her2-specific affibody, Tumor hypoxia, Radiosensitization

## Abstract

**Background:**

Solid tumor hypoxic conditions prevent the generation of reactive oxygen species (ROS) and the formation of DNA double-strand breaks (DSBs) induced by ionizing radiation, which ultimately contributes to radiotherapy (RT) resistance. Recently, there have been significant technical advances in nanomedicine to reduce hypoxia by facilitating in situ O_2_ production, which in turn serves as a “radiosensitizer” to increase the sensitivity of tumor cells to ionizing radiation. However, off-target damage to the tumor-surrounding healthy tissue by high-energy radiation is often unavoidable, and tumor cells that are further away from the focal point of ionizing radiation may avoid damage. Therefore, there is an urgent need to develop an intelligent targeted nanoplatform to enable precise enhanced RT-induced DNA damage and combined therapy.

**Results:**

Human epidermal growth factor receptor 2 (Her2)-specific dimeric affibody (Z_Her2_) mediated cisplatin-loaded mesoporous polydopamine/MnO_2_/polydopamine nanoparticles (Pt@mPDA/MnO_2_/PDA-Z_Her2_ NPs) for MRI and enhanced chemo-radiotherapy of Her2-positive ovarian tumors is reported. These NPs are biodegradable under a simulated tumor microenvironment, resulting in accelerated cisplatin release, as well as localized production of O_2_. Z_Her2_, produced using the *E. coli* expression system, endowed NPs with Her2-dependent binding ability in Her2-positive SKOV-3 cells. An in vivo MRI revealed obvious T_1_ contrast enhancement at the tumor site. Moreover, these NPs achieved efficient tumor homing and penetration via the efficient internalization and penetrability of Z_Her2_. These NPs exhibited excellent inhibition of tumor growth with X-ray irradiation. An immunofluorescence assay showed that these NPs significantly reduced the expression of HIF-1α and improved ROS levels, resulting in radiosensitization.

**Conclusions:**

The nanocarriers described in the present study integrated Her2 targeting, diagnosis and RT sensitization into a single platform, thus providing a novel approach for translational tumor theranostics.

**Graphic abstract:**

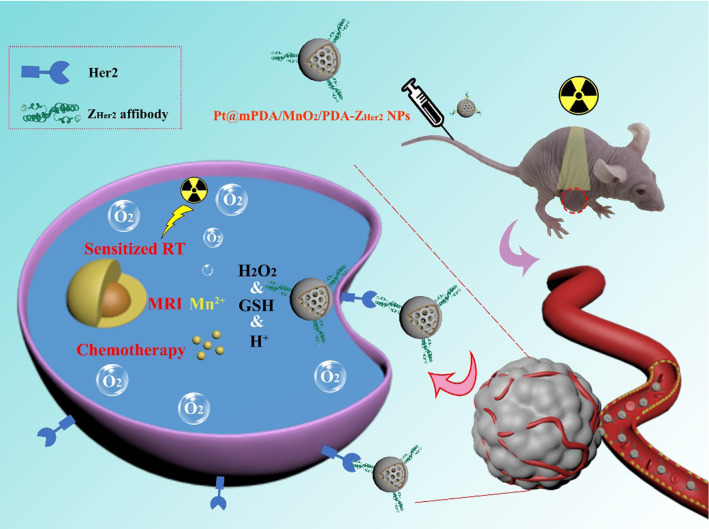

**Supplementary Information:**

The online version contains supplementary material available at 10.1186/s12951-021-00885-6.

## Background

Radiotherapy (RT), or the precise application of high energy ionizing radiation at the tumor site, can directly induce DNA breakages in tumor cells and/or indirectly damage tumor cells by generating reactive oxygen species (ROS) via water radiolysis and is an important tumor treatment strategy [1, 2]. More than 60% of malignant tumor patients receive RT at some stage during their illness, and 40% of the treatments are successful [3, 4]. However, the tumor microenvironment (TME) is complex and can contribute to the acquisition of RT resistance. Hypoxic conditions in the solid TME can be ascribed to an imbalance between the supply and consumption of O_2_ in rapidly proliferating tumor cells, as well as dysfunctional tumor vasculature [5]. The hypoxic TME prevents ROS generation and formation of DNA double-strand breaks (DSBs), which are usually induced by ionizing radiation [2, 5], ultimately leading to RT resistance. Furthermore, tumor hypoxic conditions can bring about upregulation of hypoxia-inducible factor 1α (HIF-1α), which promotes endothelial cell survival following RT and thus further promotes RT resistance [6].

Traditional medical methods have made use of hyperbaric oxygen inhalation to alleviate hypoxic conditions and improve the tumor concentration of O_2_, which acts a reservoir of radiation-induced ROS [6–8]. However, the dysfunctional tumor vascular system hinders the delivery of inhaled O_2_ to the tumor, and the potential for oxygen poisoning, barometric injury, and decompression disease seriously limits the clinical utility of this method [9]. For decades, research has focused on the enhancement of RT efficacy by increasing radiation doses, but the severe side effects caused by excessive high-energy radiation to normal tissue and organs are unavoidable [5, 10]. Recently, there have been significant technical advances in nanomedicine, such as artificial blood substitutes and nanocatalysts, which relieve hypoxia by intra-tumoral O_2_ delivery and/or in situ production of O_2_ and serve as “radiosensitizers” to treat tumor cells with low and safe doses of ionizing radiation [10, 11]. For example, Gao et al. developed a perfluorocarbon (PFC)-based nanoscale artificial red blood cell system to relieve tumor hypoxia and thus improve sensitivity to RT. With biomimetic cloaking of the red blood cell membrane, these artificial blood cells effectively delivered O_2_ into the tumor tissue, greatly relieved hypoxia, and thus remarkably improved the antitumor efficacy of RT [12]. In a recent study, Chen et al. fabricated a catalase-loaded nanoplatform for enhanced RT efficacy. The cargo catalase can trigger rapid decomposition of endogenous hydrogen peroxide (H_2_O_2_) into O_2_ to relieve tumor hypoxia and thus caused enhanced RT efficacy compared to that of X-ray radiation alone [13]. Manganese dioxide (MnO_2_) NPs provide particularly fascinating properties that have been applied for TME-responsive biodegradation, glutathione (GSH)-triggered magnetic resonance imaging (MRI) and serving as a chemodynamic therapeutic agent, which suggests that they have great potential as tumor theranostics [14–16]. Moreover, as an inorganic nano-catalyst, MnO_2_ possesses the ability to catalyze the generation of O_2_ by decomposing H_2_O_2_ at the nano level, making MnO_2_-based agents a promising candidate for improving the efficacy of O_2_-dependent therapy [17–20]. For example, Yang et al. developed a biodegradable hollow MnO_2_ nanoplatform for TME-specific MRI and drug release, modulating the hypoxic TME to enhance photodynamic therapy (PDT). Under an acidic TME, these NPs experienced rapid degradation, resulting in the on-demand release of the loaded therapeutic molecules (the photodynamic agent Ce6 and DOX) and the MRI agent Mn^2+^ and simultaneous induction of decomposition of endogenous H_2_O_2_ into O_2_ to relieve tumor hypoxia and achieve enhanced PDT efficacy [21].

Despite the superior efficacy of MnO_2_-based radiosensitizers, the off-target damage to the tumor-surrounding healthy tissue caused by high-energy radiation is often unavoidable [22]. Therefore, there is an urgent need to develop an intelligent, targeted nanoplatform as a “magic bullet” to precisely enhance RT-induced DNA damage with low side effects. One effective approach is to couple these therapeutic formulations with ligands (such as folate, Arg–Gly–Asp tripeptides, and/or hyaluronic acid) [23–25] or antibodies [26–28] that specifically recognize tumor cells or tumor vasculature-associated antigens. This would allow targeted delivery of drugs or radiosensitizers into tumor cells by ligand- or antibody-mediated enhanced endocytosis [29]. However, molecular ligands obtained using chemical approaches have limited affinity for their targets because of their simple structure, and an increasing number of studies have reported the potential immunogenicity of these synthetic agents, which severely impacts their performance [30, 31]. In addition, these agents have relatively poor thermal or chemical stability, poor tumor penetration, and slow blood clearance and are expensive to produce, severely limiting their use [32].

Affibodies, a novel class of non-immunoglobulin-based scaffold proteins, consist of a 58-amino acid residue three-helix bundle Z domain derived from one of five homologous domains (the B domain) in *Staphylococcus aureus* protein A [33]. Affibodies can specifically bind to a large range of different target proteins with high affinity by phage display of combinatorial libraries in which typically 13 side-chains on the surface of helices 1 and 2 in the Z domain have been randomized [34]. Besides the ability to bind different targets, there is no relationship between affibodies and antibodies because of having no sequence or structural homology. Possessing a small molecular weight (only ~ 6.5 kDa) and a three-helical-bundle Z domains, the affibody shows a reversible and rapid folding rate (the folding time is only 3 μs), high thermal tolerance, high specificity, and nanomolar affinities for tumors [35]. Meanwhile, their robust molecular structure endows them with high chemical tolerance, including a wide pH range (5.5–11) [36]. Moreover, affibodies do not contain disulfide bonds or free cysteines intramolecularly, which allows them to be functionally expressed in the reducing environment of the bacterial (i.e., *E. coli*) cytoplasm at high levels and low cost [37].

Human epidermal growth factor receptor 2 (Her2), which is specifically overexpressed in a significant number of ovarian, gastric and breast cancers, has become a promising target for tumor diagnosis and treatment. Affibodies binding to the extracellular domain of Her2 were previously obtained after phage display selection and these selected affibodies could bind specifically to Her2 but target a different epitope than that targeted by trastuzumab [38]. The original Her2-binding affibody molecule Z_Her2:4_ with an affinity of approximately 50 nM for Her2 was selected for further studies. Z_Her2:342_ was developed by the directed combinatorial mutagenesis at the binding site based on sequence of Z_Her2:4_ [39]. Compared to Z_Her2:4_, the Her2-binding affinity of this descendants Z_Her2:342_ increased from 50 nM to 22 pM. The Z_Her2:342_ was recently further improved by substitution of 11 amino acids in the scaffold and novel Her2 affibody molecule designated Z_Her2:2891_ was appeared. After these substitutions, Z_Her2:2891_ had higher hydrophilicity, thermal stability, diminished background interactions with immunoglobulins, full production flexibility as well as fully retained in vitro and in vivo functionality [40]. Z_Her2:2891_ has been conjugated with a DOTA moiety (denoted ABY-025) at a unique C-terminal cysteine and these affibodies have been introduced in a series of molecular imaging clinical trials [41, 42]. Thus, Z_Her2:2891_ is an ideal targeting moiety to construct Her2-targeted drugs for tumor diagnosis and treatment. However, to the best of our knowledge, no Z_Her2:2891_ mediated nano-drugs for tumor diagnosis and treatment have been reported to-date. This deficiency promoted us to investigate whether Z_Her2:2891_-directed nano-drugs could improve the efficacy of targeted tumor diagnosis and treatment.

**Scheme 1:**
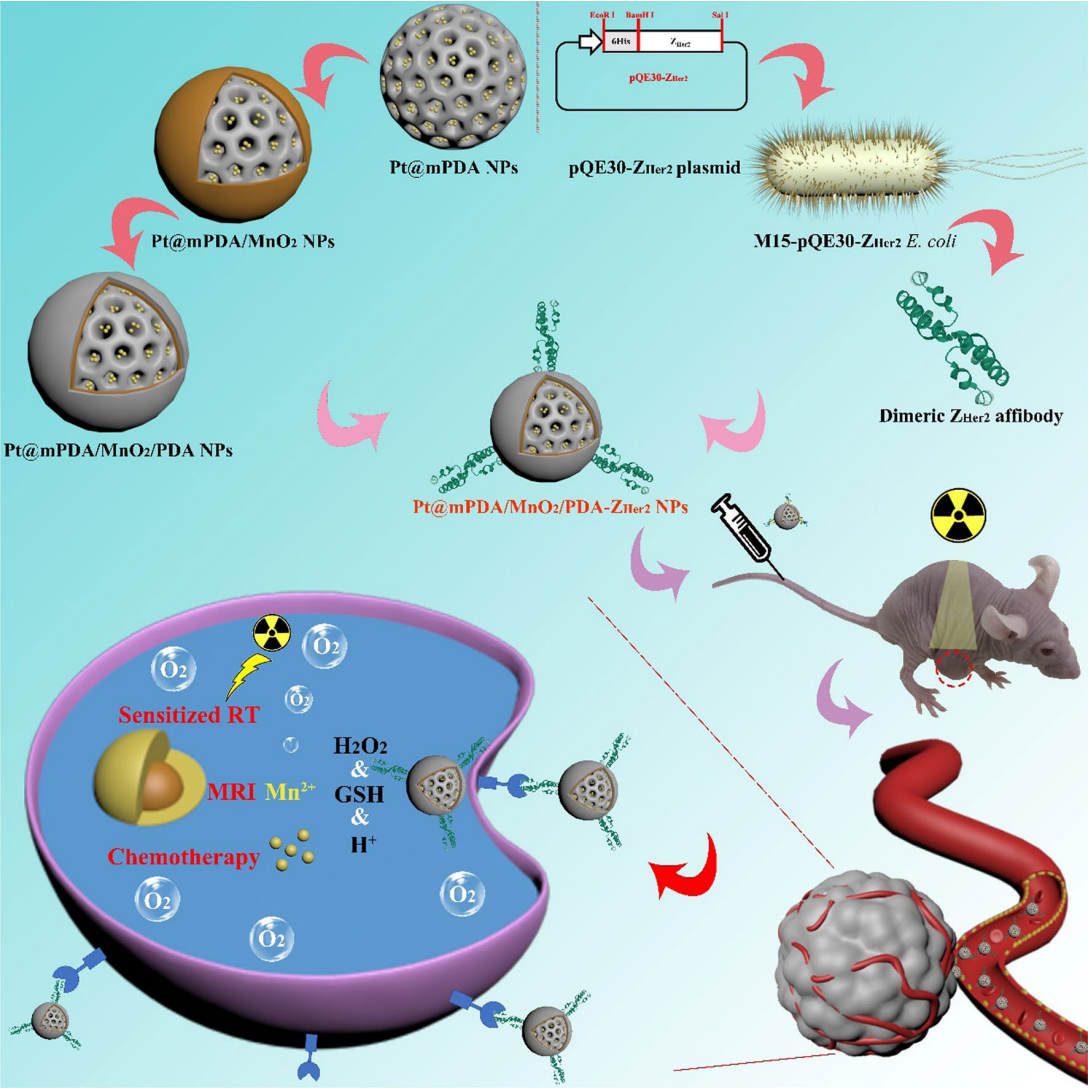
Experimental workflow for the preparation of Pt@mPDA/MnO_2_/PDA-Z_Her2_ NPs and in vivo MRI-guided enhanced chemo-radiotherapy

Herein, the dimeric Her2-specific affibody-mediated nanoparticles, denoted Pt@mPDA/MnO_2_/PDA-Z_Her2_ NPs, were developed to enhance MRI imaging and chemo-radiotherapy of Her2-overexpressing ovarian cancer. As shown in Scheme 1, mesoporous polydopamine nanoparticles (mPDA NPs) with high biocompatibility, easy face functionalization, and wet adhesion [43, 44] were first prepared by a nanoemulsion assembly method. As some tumor cells at a distance from the focal spot of ionizing radiation may avoid damage, a chemo-radiotherapy combination of mPDA NPs involving cisplatin (Pt@mPt NPs) was synthesized. Then, a thin MnO_2_ layer was grown on the peripheral surface of Pt@mPDA NPs by in situ reduction of KMnO_4_ to induce endogenous H_2_O_2_ decomposition into O_2_ and reduce tumor hypoxia. To improve the biocompatibility of the NPs and provide a reactive surface, a biomimetic PDA layer was polymerized on the surface of the MnO_2_ layer, yielding Pt@mPDA/MnO_2_/PDA NPs. Z_Her2_ affibodies were ultimately linked to the peripheral PDA layer, resulting in the final intelligent Pt@mPDA/MnO_2_/PDA-Z_Her2_ NPs. These NPs are expected to provide a suitable means to intelligently target tumor cells and sensitize them to RT, enabling the application of lower doses of radiation and decreasing the damage to the tumor-surrounding healthy tissue. This system has the potential for clinical applications in the treatment of solid tumors, which have thus far evaded the efforts of the medical and scientific community.

## Results and discussion

### Preparation and characterization of Pt@mPDA/MnO_2_/PDA NPs

**Fig. 1:**
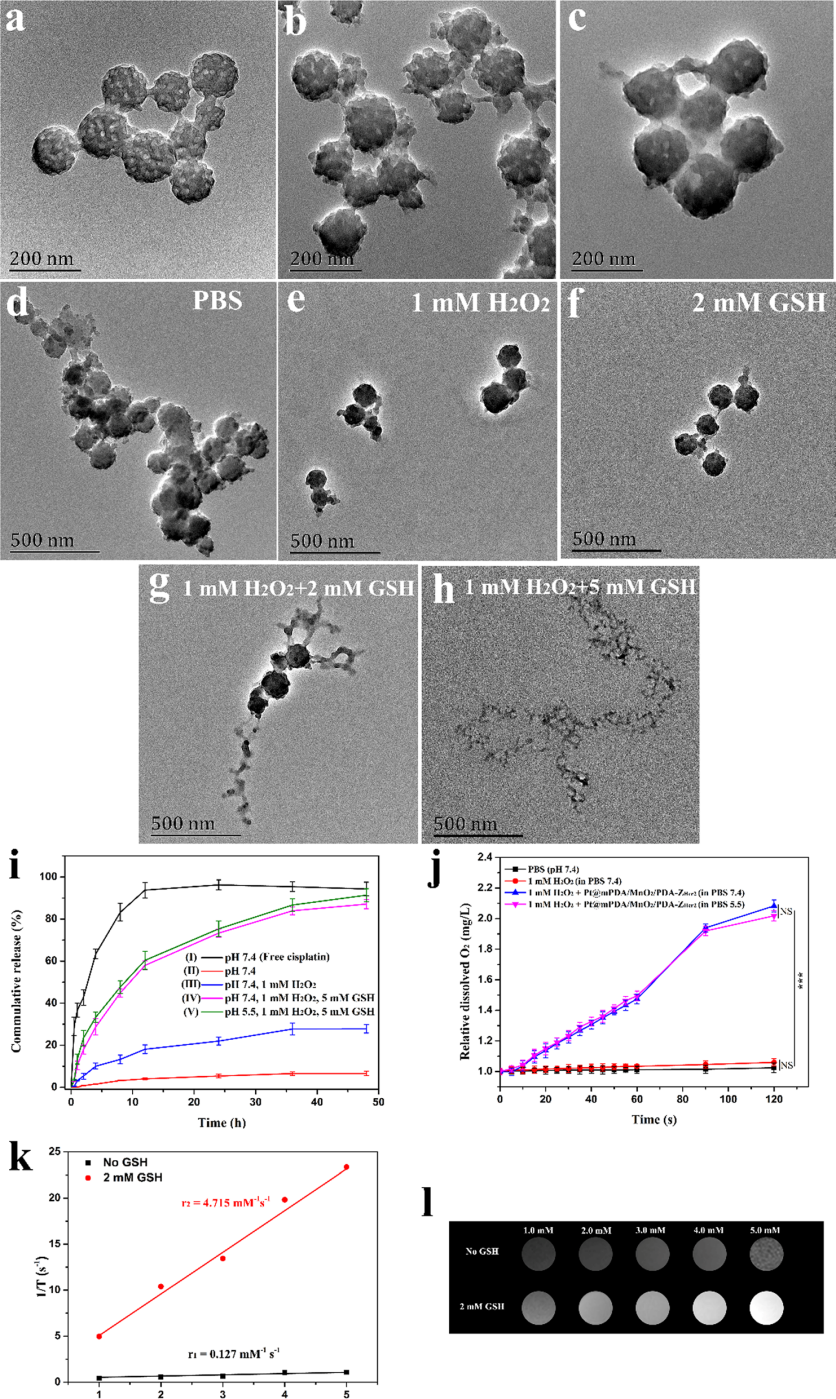
TEM images of **a** mPDA, **b** Pt@mPDA/MnO_2_, and **c** Pt@mPDA/MnO_2_/PDA NPs. **d**–**h** TEM images of Pt@mPDA/MnO_2_/PDA NPs after incubation in different buffers for 14 days. **i** The free cisplatin release behavior in PBS (I), and the release profiles of cisplatin from Pt@mPDA/MnO_2_/PDA NPs under different conditions (II–V). Data are shown as mean ± S.D. from three independent experiments. **j** The relative changes in the concentration of dissolved O_2_ in different buffers. **k** The linear fit of 1/T_1_ of mPDA/MnO_2_/PDA NPs without or with 2 mM of GSH and **l** the corresponding T_1_-weighted images with different Mn concentrations

Mesoporous PDA (mPDA) NPs were initially synthesized using a nano-emulsion assembly approach. Transmission electron microscopy (TEM) images show that the mPDA NPs are highly uniform with mesostructured morphologies (Fig. 1a) and have a mean size of 119 nm. The mesoporous shape was further characterized by N_2_ absorption–desorption. As shown in Additional file 1: Fig. S1a and 1b, the surface area and average pore diameter of the mPDA NPs were 36.84 m^2^/g and 2 nm, respectively. After cisplatin loading by electrostatic force between negatively charged mPDA and positively charged cisplatin (Additional file 1: Fig. S1c), a MnO_2_ layer was formed on the surface of Pt@mPDA NPs, which attributed to the redox reaction between reductive PDA and KMnO_4_ under neutral conditions. As shown in Fig. 1b, a thin layer on the surface of Pt@mPDA NPs is clearly observed, and the mean size increased to 139 nm. To improve the biocompatibility of the NPs and provide a chemically reactive surface for further functional modification, a biomimetic PDA layer was then polymerized on the surface of the Pt@mPDA/MnO_2_ NPs under weak alkaline conditions (pH 8.6). The mean size further increased to ~ 163 nm, and the mesoporous structure almost disappeared (Fig. 1c).

Step-wise zeta potential changes (Additional file 1: Fig. S1c) were observed, indicating the successful coating of MnO_2_ and the PDA layer on the NPs. After the final PDA layer surface functionalization, the resulting NPs were denoted Pt@mPDA/MnO_2_/PDA NPs.

The chemical characteristics of the Mn-derived NPs were further examined. Wide scan X-ray photoelectron spectroscopy (XPS) spectra (Additional file 1: Fig. S2a) of the NPs showed the characteristic peaks of C 1s, N 1s, O 1s, and Mn 2p. Mn 2p spectra (Additional file 1: Fig. S2b) showed two characteristic peaks at 653.1 eV and 641.6 eV, which confirm the Mn 2p_3/2_ and Mn 2p_1/2_ orbits of the Mn^4+^ oxidation state. These results confirm the successful deposition of MnO_2_ onto the NP surface.

The cisplatin and MnO_2_ contents of the Pt@mPDA/MnO_2_/PDA NPs were quantified by inductively coupled plasma-atomic emission spectrometry (ICP-AES) and found to be ~ 96 mg/g and ~ 164 mg/g, respectively. Taken together, these results validate the successful construction of Pt@mPDA/MnO_2_/PDA NPs.

### In vitro degradation and drug release

The degradation of NPs triggered by H_2_O_2_ and GSH was measured by determining the UV–Vis absorption of Pt@mPDA/MnO_2_/PDA NPs in different media. As shown in Fig. S3, the NPs were stable at neutral pH (Additional file 1: Fig. S3a) but degraded in an H_2_O_2_- and GSH-sensitive manner, as the absorption decreased over time in PBS containing 1 mM H_2_O_2_ or 2 mM GSH (Additional file 1: Fig. S3b, c). In striking contrast, the absorption of the dispersed NPs decreased abruptly in a mixture of 1 mM H_2_O_2_ and 2 mM GSH (Additional file 1: Fig. S3d), and this decrease was further enhanced after increasing the GSH concentration to 5 mM (Additional file 1: Fig. S3e). After 14 d, the absorbance level of Pt@mPDA/MnO_2_/PDA NPs in different media was compared. Apparently, the NPs treated with 1 mM H_2_O_2_ and 5 mM GSH showed the greatest degradation degree (Additional file 1: Fig. S3f). The morphology of the NPs after 14 d was assessed by TEM (Fig. 1d–h), correspondingly, the NPs treated with 1 mM H_2_O_2_ and 5 mM GSH showed the greatest degree of degradation, revealing H_2_O_2_- and GSH-sensitive degradation. The former was ascribed to PDA-accelerated degradation in the presence of H_2_O_2_ [45], while the latter was attributed to the redox reaction between the MnO_2_ layer and GSH [14, 15]. These results suggest that the prepared mPDA/MnO_2_/PDA NPs are promising biodegradable materials for TME-specific drug release and in vivo stability.

The release profiles of free cisplatin in PBS (pH 7.4) were firstly measured. The free cisplatin gave a burst of drug release in initial 30 min of the experiment, and reached a cumulation release of ~ 93% after 12 h (Fig. 1i (I)). In contrast, the Pt@mPDA/MnO_2_/PDA NPs exhibited H_2_O_2_, GSH, and pH triple model-responsive drug release (Fig. 1i (II–V)). In blank PBS (pH 7.4), cisplatin was released slowly, with only ca. ~ 6.6% of the incorporated cisplatin released after 48 h, while a more rapid and extensive drug release (ca. ~ 27.8% after 48 h) occurred after supplementing with 1 mM H_2_O_2_. In contrast, drug release occurred to a greater extent (ca. ~ 88%) after the addition of 5 mM GSH. These data support the observations of NP degradation under simulated TME conditions. Because of the pH-triggered degradation of MnO_2_ [21], it was noted that the drug release rate was further accelerated under acidic conditions (pH 5.5). In summary, Pt@mPDA/MnO_2_/PDA NPs exhibit drug release under TME-specific conditions, thus potentially reducing off-target drug leakage.

### Decomposition of H_2_O_2_ triggered by NPs and detection of ROS in vitro

The solid tumor hypoxic microenvironment severely limits the efficacy of RT because the DNA damage and ROS generation induced by ionizing radiation are prevented under hypoxic conditions [5, 6]. To overcome this problem, NPs containing MnO_2_ were constructed in order to catalyze the decomposition of H_2_O_2_ to O_2_. Significant and sustained amounts of O_2_ were generated after adding mPDA/MnO_2_/PDA NPs ([MnO_2_] = 2 μg/mL) to PBS (pH 7.4 and 5.5) containing H_2_O_2_, while PBS with or without H_2_O_2_ in the absence of NPs maintained a stable O_2_ concentration (Fig. 1j).

A MB degradation method was used to examine the ROS generation. As shown in Additional file 1: Fig. S4, a significant decline in MB absorbance was observed after treating X-ray (6 Gy), demonstrating that the ROS generation via water molecule radiolysis had occurred. Addition of MnO_2_-free Pt@mPDA/PDA NPs did not increase the MB degradation level compared to X-ray treatment, while Pt@mPDA/MnO_2_/PDA NPs without X-ray treatment had negligible effect on MB degradation. However, for the MnO_2_-containing Pt@mPDA/MnO_2_/PDA NPs and X-ray combination, the MB degradation level was increased significantly. These results confirm that the MnO_2_-containing NPs can enhance radiation-induced ROS generation.

### GSH-activated MRI

The GSH-activated T_1_-MRI contrast performance of mPDA/MnO_2_/PDA NPs was investigated with and without GSH. As expected, the NPs showed much stronger T_1_-weighted MR signal enhancement upon exposure to GSH with a 37.1-fold increase in the r_1_ value compared to the NPs in the absence of GSH (Fig. 1k). Correspondingly, a concentration-dependent brightening effect of mPDA/MnO_2_/PDA NPs upon treatment with GSH was observed (Fig. 1l), which can be attributed to the GSH-activated MRI agent Mn^2+^. These results confirm that the MnO_2_-functionalized NPs are promising candidates for tumor-specific imaging.

### Preparation and characterization of Pt@mPDA/MnO_2_/PDA-Z_Her2_ NPs

**Fig. 2:**
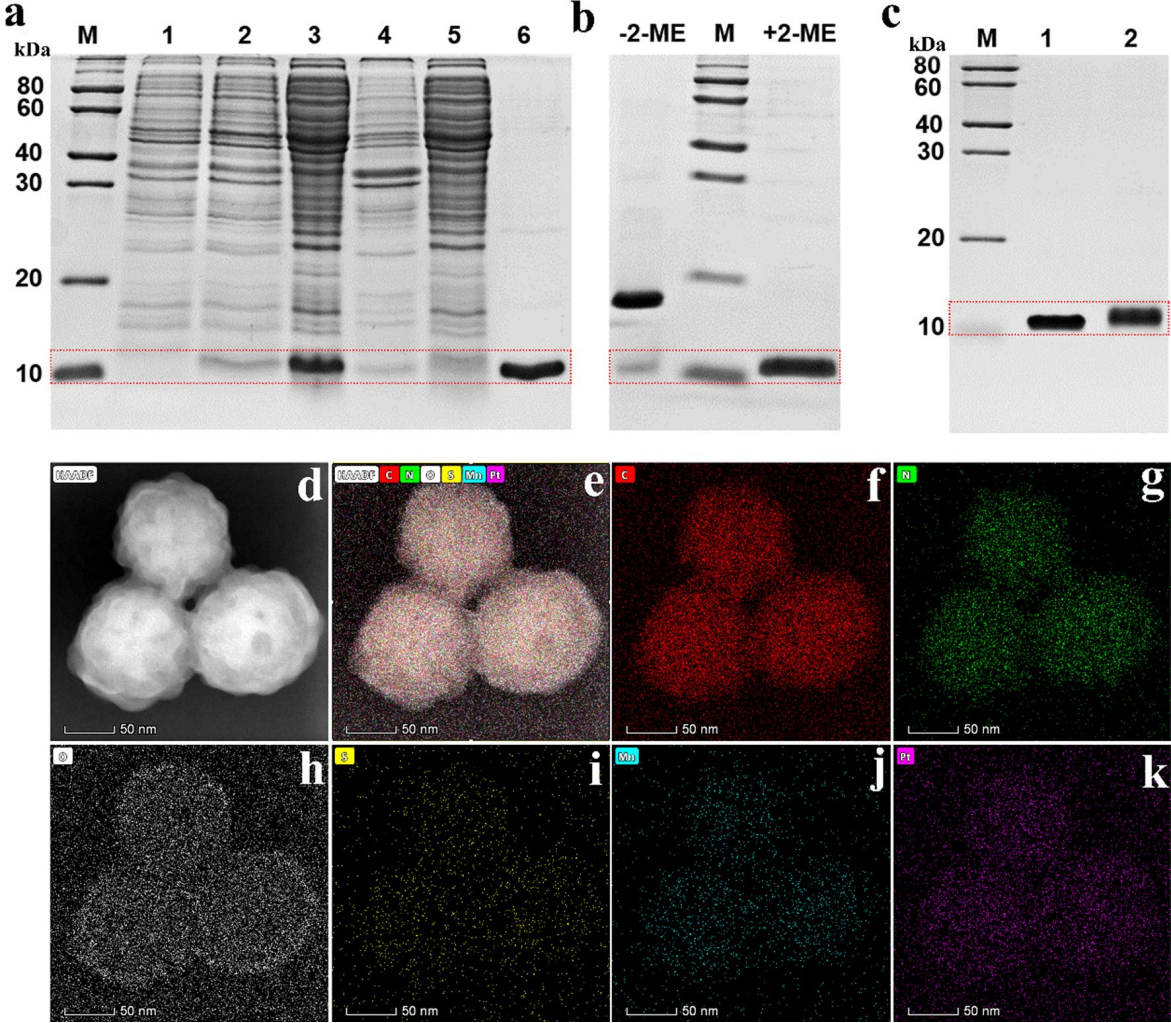
**a** SDS-PAGE of samples collected during Z_Her2_ preparation. M, protein marker; lane 1, total protein of uninduced cells; lane 2, total protein of induced cells; lane 3, soluble protein of induced cells; lane 4, insoluble protein of induced cells; lane 5, flow-through fractions after binding to Ni–NTA resin; lane 6, protein purified by Ni–NTA affinity chromatography. **b** SDS-PAGE of the purified Z_Her2_ affibody in the presence or absence of 2-ME. **c** SDS-PAGE characterization of Z_Her2_ labelled with 6-FAM. M, protein marker; lane 1, unlabelled Z_Her2_; lane 2, 6-FAM-labelled Z_Her2_. **d** HAADF-STEM images of Pt@mPDA/MnO_2_/PDA-Z_Her2_ NPs. Element mapping of Pt@mPDA/MnO_2_/PDA-Z_Her2_ NPs: **e** merged image, **f** C, **g** N, **h** O, **i** S, **j** Mn, and **k** Pt

Since polymerization might improve the stability and avidity of affibodies [46], we first prepared a novel dimeric Her2-binding affibody designated Z_Her2_ (amino acid sequence: mrgshhhhhhgsaeakyakemrnayweiallpnltnqqkrafirklyddpsqssellseakklndsqapkc) based on Z_Her2:2891_ through disulfide bond formed by the addition of a cysteine at the C-terminus. The Z_Her2_ affibody was expressed by the *E. coli* expression system and purified using Ni–NTA affinity chromatography. The expected molecular weight of the affibody is approximately 8 kDa. Compared to the protein isolated from cells before induction (Fig. 2a, lane 1), a protein with a molecular weight of approximately 10 kDa was induced by IPTG (Fig. 2a, lane 2). Following cell disruption, SDS-PAGE analysis of the total protein in the soluble (Fig. 2a, lane 3) and insoluble (Fig. 2a, lane 4) fractions revealed that the recombinant protein was mainly expressed as a soluble protein. After binding to Ni–NTA affinity resin, the amount of recombinant protein in the flow-through fractions was remarkably reduced (Fig. 2a, lane 5), suggesting high purification efficiency. The purified Z_Her2_ protein was visualized as a single protein band on an SDS-PAGE gel, and the purity was calculated to be more than 95% according to scanning densitometry of the electrophoretic bands (Fig. 2a, lane 6). Approximately 30–40 mg of protein was obtained from *E. coli* cells in 1 L of culture.

The affinity of the dimeric form affibody for its target is usually higher than that of the monomeric form [47]. So, the Z_Her2_ affibody was dimerized upon the addition of a cysteine residue at the C-terminus. As expected, the molecular weight of Z_Her2_ under natural conditions (Fig. 2b, in the absence of 2-ME) was approximately double than that of Z_Her2_ under reductive conditions (Fig. 2b, in the presence of 2-ME), indicating that Z_Her2_ forms disulfide bonds and dimers under natural conditions.

A slight increase in molecular weight was observed after Z_Her2_ was labelled with the fluorescent agent 6-Carboxfluorescein (6-FAM) (Fig. 2c), with a labelling efficiency was approximately 100%. This label allowed for visual evaluation of the binding capacity between FAM-Z_Her2_ and Her2-positive cells.

Finally, the Z_Her2_ affibody was coupled with Pt@mPDA/MnO_2_/PDA NPs via a Michael addition/Schiff base reaction by conjugating the amino group to the oxidized quinone form of the catechol groups under weak alkaline conditions. The mean hydrodynamic size of NPs increased slightly from 185 to 201 nm after Z_Her2_ affibody conjugation (Additional file 1: Fig. S5a). Both NPs exhibited high colloidal stability and could be dispersed in PBS without aggregation over 24 h (Additional file 1: Fig. S5b). The elemental mapping images (Fig. 2d–k) of mPDA/MnO_2_/PDA-Z_Her2_ NPs showed a homogeneous distribution of C, N, O, S, Mn, and Pt, further confirming the successful loading of cisplatin and coupling of Z_Her2_. In addition, the Z_Her2_ content was approximately 0.8 mg/g according to ICP-AES quantitation of the Z_Her2_-specific S element.

### Her2-positive cell-specific binding and cytotoxicity assay

Flow cytometry (FCM) and confocal laser scanning microscopy (CLSM) (Additional file 1: Fig. S6a–c) showed a concentration-dependent increase in fluorescence for a human ovarian cancer cell line (SKOV-3) after incubation with FITC-labelled anti-Her2 antibodies, while negligible FITC fluorescence was visible in a breast cancer cell line (MCF-7). This confirmed that SKOV-3 cells are Her2-positive, while MCF-7 cells are Her2-negative, which is consistent with previous results [48]. The binding activity of FAM-Z_Her2_ with Her2-negative MCF-7 cells and Her2-positive SKOV-3 cells was examined by CLSM. As shown in Additional file 1: Fig. S7a, FAM-Z_Her2_ specifically binds to SKOV-3 cells, while negligible FAM-Z_Her2_ binding to MCF-7 cells was seen, indicating that these affibodies bind specifically to Her2-positive cancer cells. This Her2-specific binding activity was further confirmed by FCM, as strong FAM fluorescence was visible in SKOV-3 cells, while the binding was significantly reduced after pre-incubation with free Z_Her2_ (Additional file 1: Fig. S7b).

FCM analysis was performed to evaluate the affinity of affibody monomers and dimers to Her2 receptors by measuring the FAM fluorescence intensity. As shown in Additional file 1: Fig. S8a, the binding rates of the dimeric Her2 affibody were 58.4%, compared to 33.8% for the monomer Her2 affibody at the same molar concentration. This confirms that the affinity of the dimeric Her2 affibody was higher than that of the monomeric Her2 affibody for Her2 receptors.

**Fig. 3:**
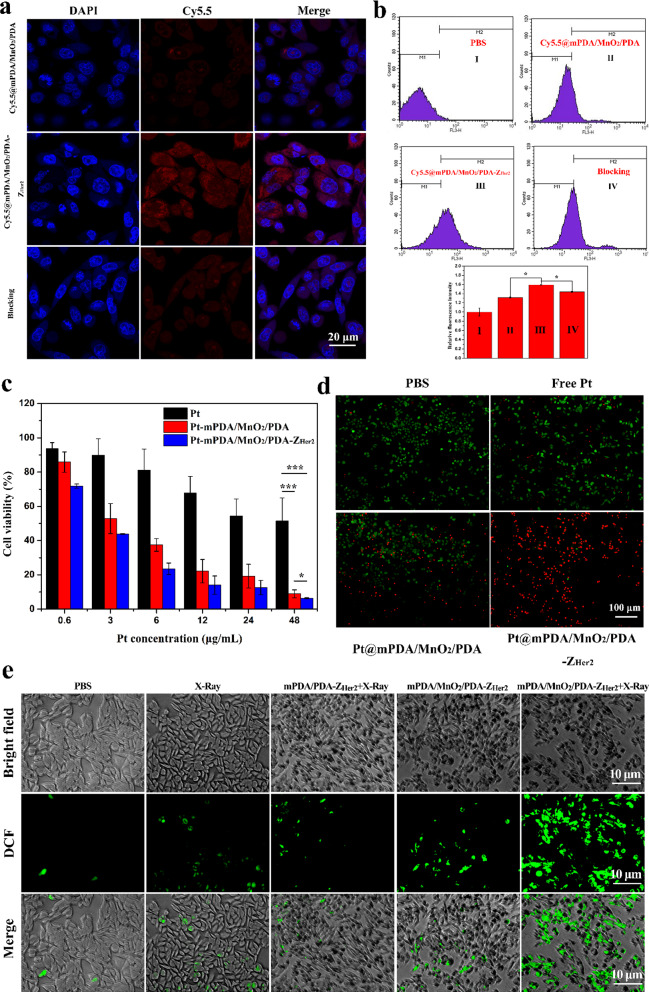
**a** CLSM images of SKOV-3 cells after 2 h incubation with Cy5.5@mPDA/MnO_2_/PDA NPs and Cy5.5@mPDA/MnO_2_/PDA-Z_Her2_ NPs and after a 1 h pre-treatment with or without Z_Her2_. **b** Flow cytometry data for untreated SKOV-3 cells, SKOV-3 cells incubated for 4 h with Cy5.5@mPDA/MnO_2_/PDA NPs or Cy5.5@mPDA/MnO_2_/PDA-Z_Her2_ NPs, and cells pre-incubated with Z_Her2_ for 1 h before being exposed to Cy5.5@mPDA/MnO_2_/PDA-Z_Her2_ NPs for 4 h, and fluorescence intensity quantified. **c** MTT viability results for SKOV-3 cells after incubation with free cisplatin (Pt), Pt@mPDA/MnO_2_/PDA NPs, and Pt@mPDA/MnO_2_/PDA-Z_Her2_ NPs for 24 h. **d** Fluorescence images of calcein-AM/PI co-stained SKOV-3 cells after different treatments (dose of cisplatin: 48 μg/mL). **e** Intracellular ROS levels in SKOV-3 cells after treatment with different formulations

To determine whether Z_Her2_ could enhance the internalization of NPs into Her2-overexpressing tumor cells, the uptake of Cy5.5-labelled mPDA/MnO_2_/PDA (Cy5.5@mPDA/MnO_2_/PDA) and mPDA/MnO_2_/PDA-Z_Her2_ (Cy5.5@mPDA/MnO_2_/PDA-Z_Her2_) NPs by SKOV-3 cells was measured using CLSM and quantified via a FCM assay. Minimal intracellular red fluorescence was observed in SKOV-3 cells after treatment with Cy5.5@mPDA/MnO_2_/PDA NPs (Fig. 3a). In striking contrast, strong Cy5.5 (red) fluorescence was visible in SKOV-3 cells after incubation with Cy5.5@mPDA/MnO_2_/PDA-Z_Her2_ NPs. A blocking experiment was carried out by pre-incubating SKOV-3 cells with free Z_Her2_ (20 μg/mL) to further explore the mechanism of endocytosis. CLSM images showed an attenuated fluorescence signal inside pre-incubated SKOV-3 cells, indicating that the occupation of Her2 receptors resulted in reduced endocytosis and therefore failed to bind the NP-conjugated Z_Her2_. Similarly, the FCM data showed that the uptake of cellular NPs significantly increased after conjugating Z_Her2_ to Cy5.5@mPDA/MnO_2_/PDA NPs; however, the cellular uptake was significantly reduced after pre-incubation of SKOV-3 cells with free Z_Her2_ (Fig. 3b). Collectively, these results confirm that the Z_Her2_ affibody can enhance the internalization of NPs into Her2-positive cancer cells due to the specific affinity between the Z_Her2_ affibody and the Her2 receptor.

The cytocompatibility of drug-free mPDA/MnO_2_/PDA-Z_Her2_ NPs with human umbilical vein endothelial cells (HUVECs) was assessed using the MTT assay. As shown in Additional file 1: Fig. S8b, negligible toxicity to non-cancerous cells was observed, even when the concentration of mPDA/MnO_2_/PDA-Z_Her2_ NPs reached 250 μg/mL (> 90% viability), thus demonstrating the good cytocompatibility of the carrier materials.

All cisplatin-containing formulations exhibited dose-dependent cytotoxicity to SKOV-3 cells (Fig. 3c). The half maximal inhibitory concentrations (IC_50_) of free cisplatin, Pt@mPDA/MnO_2_/PDA NPs, and Pt@mPDA/MnO_2_/PDA-Z_Her2_ NPs for SKOV-3 cells were 9.79 ± 0.8 μg/mL, 3.18 ± 0.3 μg/mL, and 2.56 ± 0.1 μg/mL, respectively. In contrast to free cisplatin, the Pt@mPDA/MnO_2_/PDA NPs exhibited more potent toxicity to cells over the entire cisplatin dose range (0.6–48 μg/mL), which can be ascribed to more efficient cellular uptake of NPs. More encouragingly, the conjugation of Z_Her2_ to Pt@mPDA/MnO_2_/PDA NPs further improved the cytotoxicity of the NPs because of more efficient internalization mediated by the targeted affinity for Z_Her2_ on the NPs to Her2 receptors on SKOV-3 cells.

Calcein-AM/PI double staining was performed to evaluate the degree of apoptosis of the cells. The optimal degree of cell apoptosis was observed upon treatment with Pt@mPDA/MnO_2_/PDA-Z_Her2_ NPs (Fig. 3d), which is consistent with the MTT results.

Since O_2_ is the ROS-generating resource induced by X-ray treatment, we next evaluated the effect of MnO_2_-containing NPs on the intracellular oxidative stress levels. As shown in Fig. 3e, compared to X-ray treatment alone, treatment with MnO_2_-free mPDA/PDA-Z_Her2_ NPs combined with X-ray did not increase the intracellular ROS levels, as low DCF fluorescence was observed in cells receiving X-ray treatment with or without mPDA/PDA-Z_Her2_ NPs. The intracellular oxidative stress levels of SKOV-3 cells improved after treatment with mPDA/MnO_2_/PDA-Z_Her2_ NPs alone, which can be ascribed to their ability of MnO_2_ to produce HO• (one ROS species) [14, 15]. In stark contrast, cells receiving mPDA/MnO_2_/PDA-Z_Her2_ NPs and X-ray combined treatment largely increased the intracellular ROS levels compared with cells receiving MnO_2_-free mPDA/PDA-Z_Her2_ NPs + X-ray treatment. This finding indicates that treatment with MnO_2_-containing NPs can facilitate ROS generation induced by ionizing radiation, confirming that these NPs have the capacity to increase radiosensitivity.

### Hemolysis assay in vitro

A hemolysis assay was performed to determine the pharmacological safety of the NPs. No morphological changes and no significant lysis were observed after RBCs were incubated with Pt@mPDA/MnO_2_/PDA NPs or Pt@mPDA/MnO_2_/PDA-Z_Her2_ NPs (Additional file 1: Fig. S9a, b). Both Pt@mPDA/MnO_2_/PDA NPs and Pt@mPDA/MnO_2_/PDA-Z_Her2_ NPs had low hemolytic activity with only ~ 4.4% and ~ 4.7% RBC lysis, respectively (Additional file 1: Fig. S9c). The results indicate that both Pt@mPDA/MnO_2_/PDA NPs and Pt@mPDA/MnO_2_/PDA-Z_Her2_ NPs are blood compatible biomaterials.

### Tumor targeting profiles in vivo

**Fig. 4:**
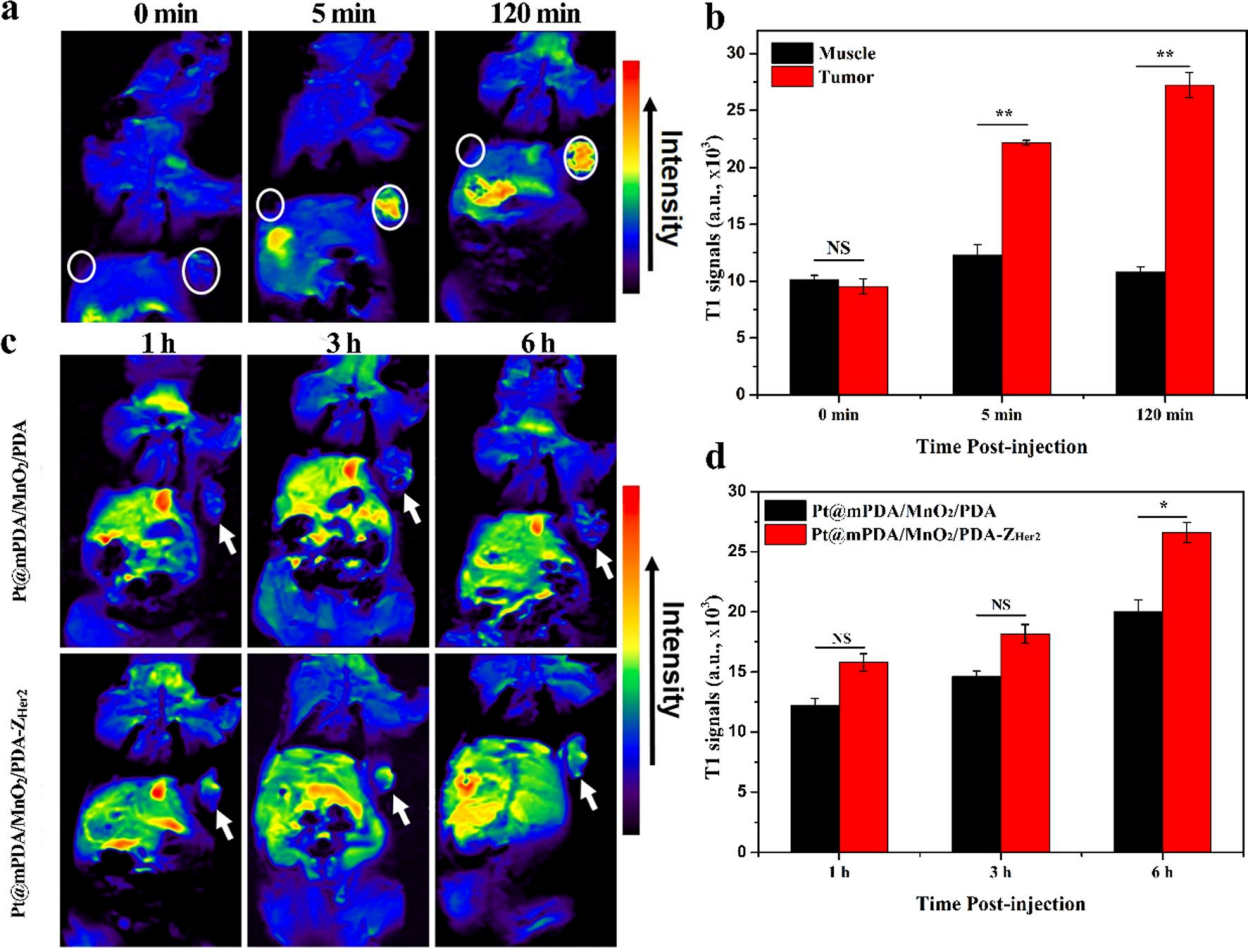
**a** T_1_-MR images of SKOV-3 tumor-bearing mice before and after direct injection of Pt@mPDA/MnO_2_/PDA NPs (50 μL in PBS; [Mn] = 50 mM) into tumor (right, circle indicated) and muscle (left, circle indicated) tissues; **b** The quantified T_1_-MR signals corresponding to (**a**); **c** T_1_-weighted images of mice bearing SKOV-3 tumor grafts (arrow indicated) intravenously injection with Pt@mPDA/MnO_2_/PDA NPs or Pt@mPDA/MnO_2_/PDA-Z_Her2_ NPs ([Mn] = 3 mM, 100 μL in PBS per mouse) at 1 h, 3 h and 6 h, respectively; **d** The quantified T_1_-MR signals corresponding to (**c**)

Based on the in vitro results, Z_Her2_-containing NPs were expected to target Her2-overexpressing tumor in vivo. The TME-activated MRI ability of NPs was first investigated in the SKOV-3 tumor-bearing mouse model. The T_1_-weighted MRI signal in the tumors increased gradually over time, while negligible MRI signal enhancement was observed in the muscles (Fig. 4a and b). This can be attributed to the reduction of the MnO_2_ layer to the MRI agent Mn^2+^ by the high levels of GSH in the TME [15, 49], which makes the MnO_2_-containing NPs particularly attractive for tumor-specific imaging applications. Tumor accumulation of Pt@mPDA/MnO_2_/PDA and Pt@mPDA/MnO_2_/PDA-Z_Her2_ NPs in SKOV-3 tumor-bearing mice at different times after intravenous injection was evaluated by tracking the MRI signal. The MRI signal was observed at the tumor site at 1 h post-injection of Pt@mPDA/MnO_2_/PDA-Z_Her2_ NPs, and the intensity increased gradually over time, indicating tumor-specific accumulation of these NPs (Fig. 4c). In contrast, decreased tumor accumulation of Pt@mPDA/MnO_2_/PDA NPs was observed as the signal of the Pt@mPDA/MnO_2_/PDA-Z_Her2_ NPs was significantly stronger than that of Pt@mPDA/MnO_2_/PDA NPs (p < 0.05) at 6 h (Fig. 4d). The biodistribution of free cisplatin, Pt@mPDA/MnO_2_/PDA, and Pt@mPDA/MnO_2_/PDA-Z_Her2_ NPs in SKOV-3 tumors was determined by quantifying the Pt content using ICP-AES at 12 h post-injection (Additional file 1: Fig. S10). The Pt content in the tumors of mice treated with Pt@mPDA/MnO_2_/PDA-Z_Her2_ NPs was approximately 3.5-fold and two fold higher than that in mice treated with free cisplatin and Pt@mPDA/MnO_2_/PDA NPs, respectively. The results reflect the more tumor retention of cisplatin-loaded NPs and tumor-targeting ability of the Z_Her2_ affibody.

**Fig. 5:**
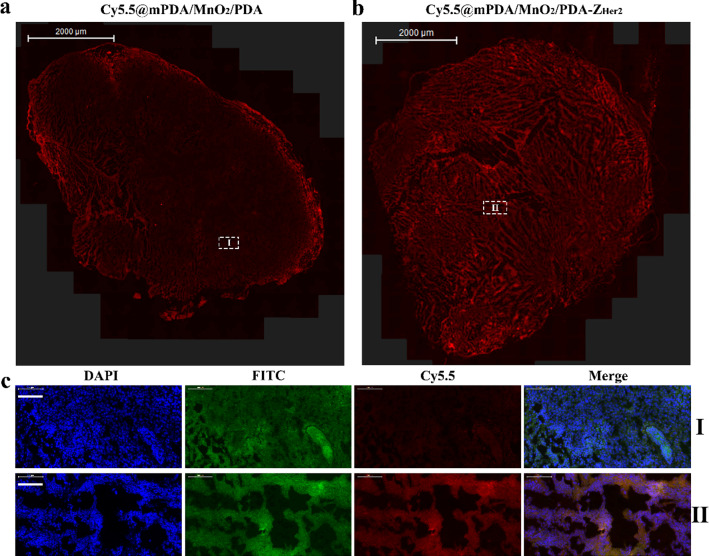
Immunofluorescence assay of Cy5.5@mPDA/MnO_2_/PDA NPs and Cy5.5@mPDA/MnO_2_/PDA-Z_Her2_ NPs in SKOV-3 tumor tissues. Mice bearing SKOV-3 tumors were intravenously injected with Cy5.5@mPDA/MnO_2_/PDA NPs or Cy5.5@mPDA/MnO_2_/PDA-Z_Her2_ NPs (100 μL; 1 mg/mL in PBS). Tumors were collected and further studied by immunofluorescence assay after 12 h post-injection. **a** The immunofluorescence images of Cy5.5@mPDA/MnO_2_/PDA NPs (red) in tumor tissues; **b** The immunofluorescence images of Cy5.5@mPDA/MnO_2_/PDA-Z_Her2_ NPs (red) in tumor tissues; **c** Representative magnified immunofluorescence images corresponding to (**a**) and (**b**). Scale bar = 100 μm. The nuclei and Her2 were stained with DAPI (blue), anti-Her2 antibody (green), respectively

Immunofluorescence and bio-TEM assays were performed to further evaluate the tumor-targeting ability of Cy5.5@mPDA/MnO_2_/PDA-Z_Her2_ NPs at the histological level. Immunofluorescence staining showed that Cy5.5 fluorescence (representing Cy5.5@mPDA/MnO_2_/PDA NPs) was mainly restricted to the tumor peripheral tissue (Fig. 5a and c (I)), suggesting poor penetration of the NPs into the TME. It was very encouraging to see that in the presence of Z_Her2_, the NPs overcame these biological barriers and penetrated deeply into the tumor tissue. As shown in Fig. 5b and c (II), strong red fluorescence was observed throughout the tumor, which co-localized with green fluorescence (representing Her2). These results confirm the efficient tumor homing and penetration of Cy5.5@mPDA/MnO_2_/PDA-Z_Her2_ NPs, which is afforded by Z_Her2_.

**Fig. 6:**
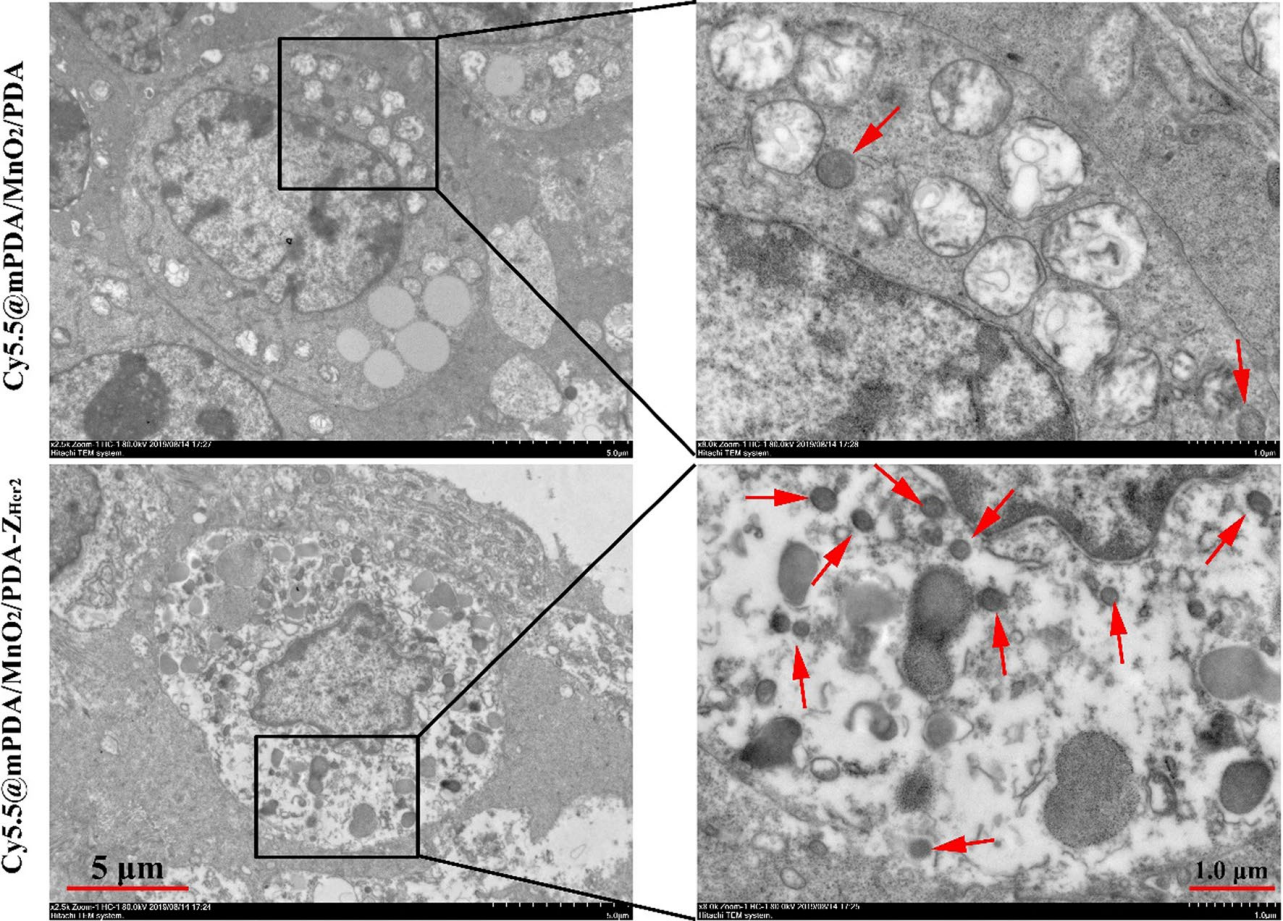
Bio-TEM images of Cy5.5@mPDA/MnO_2_/PDA NPs and Cy5.5@mPDA/MnO_2_/PDA-Z_Her2_ NPs in SKOV-3 tumor tissues. The red arrows indicate the NPs. Mice bearing SKOV-3 tumors were intravenously injected with Cy5.5@mPDA/MnO_2_/PDA NPs or Cy5.5@mPDA/MnO_2_/PDA-Z_Her2_ NPs (100 μL; 1 mg/mL in PBS). Tumors were collected and further studied by bio-TEM after 12 h post-injection

This effect was also confirmed by bio-TEM imaging (Fig. 6) of tumor tissue. Greater amounts of Cy5.5@mPDA/MnO_2_/PDA-Z_Her2_ NPs were present within the cytoplasm, with little Cy5.5@mPDA/MnO_2_/PDA NPs being observed in the tumor tissue. This superior tumor penetration and targeting capabilities are crucial for enhancing therapeutic efficacy. All these findings suggest that Z_Her2_ modification combined with a TME-triggered off-to-on diagnostic agent make Pt@mPDA/MnO_2_/PDA-Z_Her2_ NPs particularly attractive for MRI-guided tumor-targeting treatment.

### In vivo chemo-sensitized radiotherapy

**Fig. 7:**
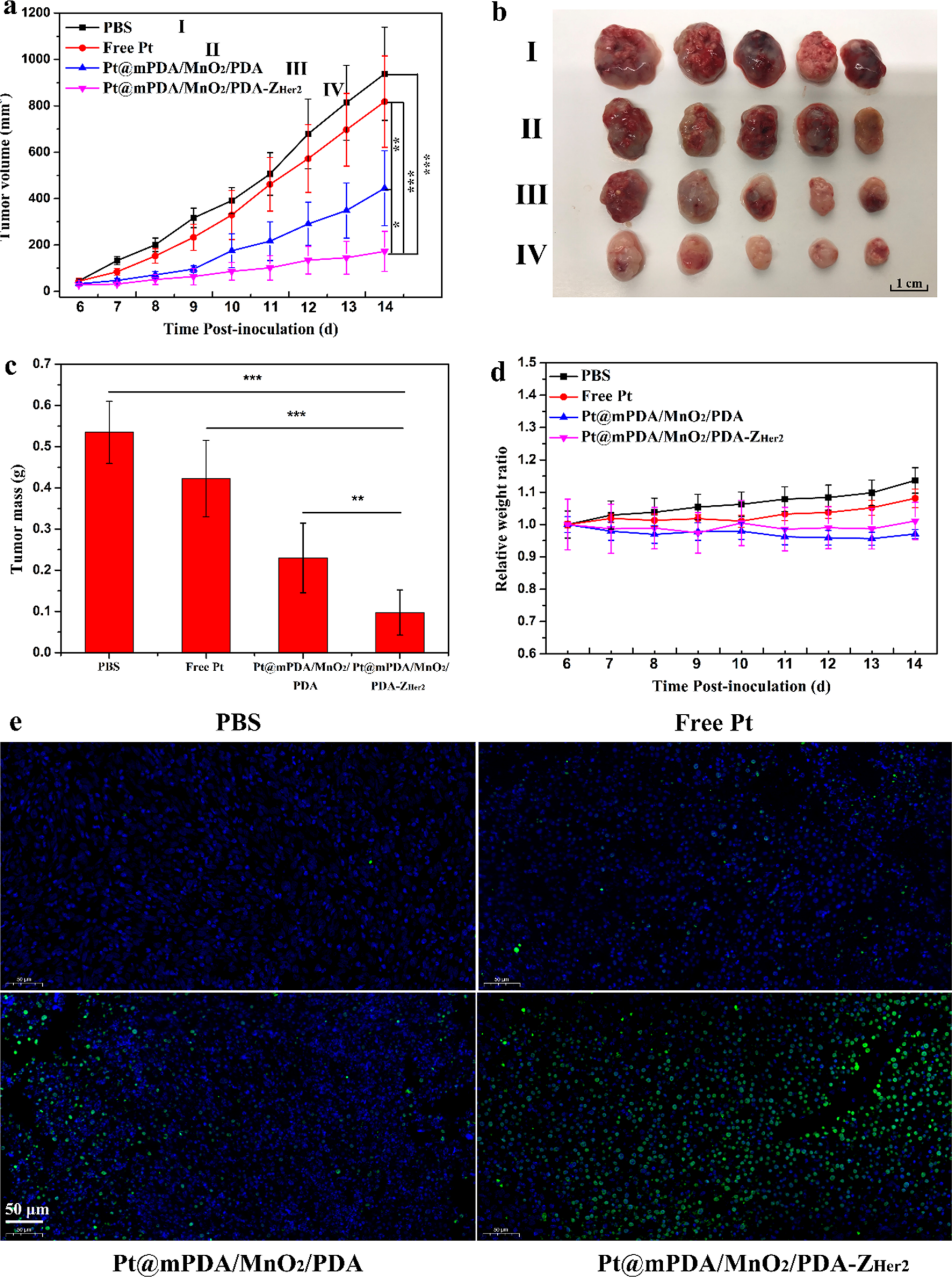
In vivo chemotherapy efficacy of different formulations in mouse bearing SKOV-3 tumor grafts. When the volume of tumors reached about 50 mm^3^, mice were intravenously injected with cisplatin, Pt@mPDA/MnO_2_/PDA NPs or Pt@mPDA/MnO_2_/PDA-Z_Her2_ NPs (dose of cisplatin = 2 mg/kg) every 2 days. Tumor-bearing mice intravenously injected with the same volume of PBS were used as control group. The tumor volume and body weight were measured every day. On the 14^th^ day after inoculation, all the tumor grafts were removed, weighed and analyzed by TUNEL staining. **a** SKOV-3 tumor growth curves of different groups after intravenously injection of the formulations. **b** The images of tumors isolated after treatment for 8 days. **c** Mean weights of the tumors isolated on day 14. **d** Body weight changes over the 8 days of the experiments. **e** TUNEL-stained images of tumor slices excised from each treatment group on day 14. The nuclei of cells were visualized using DAPI (blue)

Encouraged by the tumor-targeting and penetration effects of Z_Her2_-functionalized NPs, the in vivo chemotherapeutic activities in SKOV-3 tumor-bearing mice were then assessed. In mice treated with PBS, the tumor volume increased steadily during the treatment period (Fig. 7a). Mice that were administered free cisplatin did not exhibit appreciable tumor suppression, probably due to insufficient accumulation of cisplatin in the tumors. In sharp contrast, mice that received Pt@mPDA/MnO_2_/PDA NPs showed inhibited tumor progression as a result of the improved tumor retention of cisplatin-containing NPs. As expected, with the guidance of the Z_Her2_ affibody, Pt@mPDA/MnO_2_/PDA-Z_Her2_ NPs achieved the most potent inhibition of tumor growth, and by the end of the treatment, these extracted tumors had the smallest volume and mass (Fig. 7b and c), persuasively demonstrating the excellent targeted antitumor activity of Pt@mPDA/MnO_2_/PDA-Z_Her2_ NPs. Body weight was monitored every day, and no obvious difference was observed among all the treated groups (Fig. 7d).

A TUNEL assay was conducted to determine the levels of apoptosis in the tumor tissue. The largest number of green-colored cells (indicating the highest levels of apoptosis) were observed in tumors taken from mice receiving Pt@mPDA/MnO_2_/PDA-Z_Her2_ NPs (Fig. 7e), indicating that these NPs had the most potent antitumor effects.

**Fig. 8:**
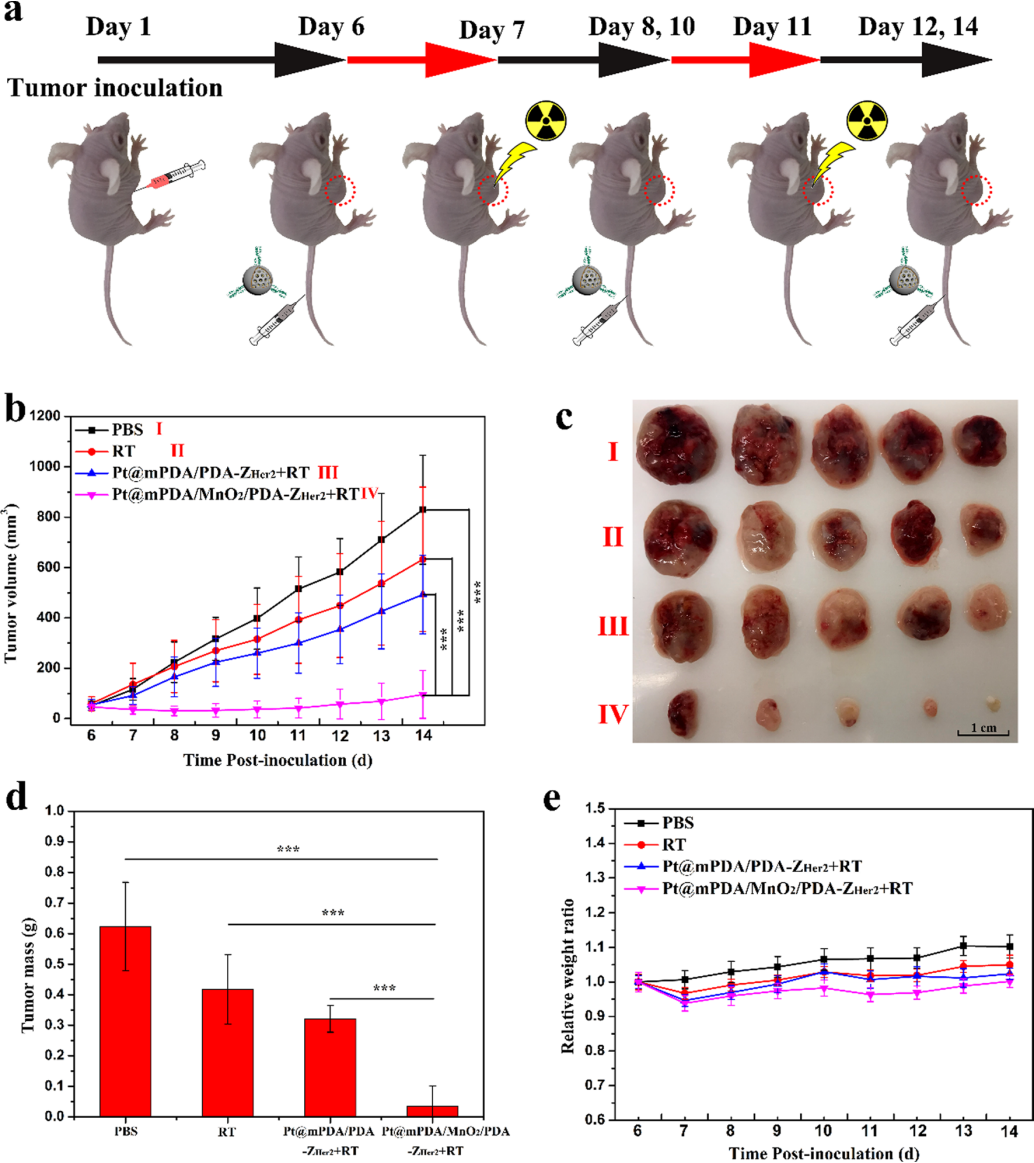
In vivo chemo-radiation combined therapy efficacy of different formulations in mice bearing SKOV-3 tumor grafts. When the volume of tumors reached about 50 mm^3^, mice were intravenously injected with MnO_2_-free Pt@mPDA/PDA-Z_Her2_ NPs, Pt@mPDA/MnO_2_/PDA NPs or Pt@mPDA/MnO_2_/PDA-Z_Her2_ NPs every 2 days. Mice in control group were intravenously injected with same volume of PBS. Mice received an X-Ray radiation at a dose of 6 Gy for 24 h post-injection every 4 days except for PBS group. Tumor sizes and body weights were recorded every day. On the 14^th^ day after inoculation, all the tumor grafts were removed, weighed. **a** Schematic illustration of process of the chemo-radiation combined therapy; **b** SKOV-3 tumor growth curves of different groups after intravenously injection of the formulations; **c** The images of tumors isolated after treatment for 8 days; **d** Mean weights of the tumors isolated on day 14; **e** Body weight changes over the 8 days of the experiments

Based on the remarkable tumor inhibition effects of Pt@mPDA/MnO_2_/PDA-Z_Her2_ NPs, the combined chemo-radiation therapeutic efficacy with these NPs, especially the enhanced RT effect, by adopting MnO_2_ as a radiosensitizer for hypoxic tumors was next assessed. The treatment schedule for chemo-radiotherapy is illustrated in Fig. 8a. RT alone did not have any significant antitumor effects, possibly because of RT resistance caused by the hypoxic TME and a sharp growth in tumor size (Fig. 8b–d) was observed during the treatment period. Mice receiving Pt@mPDA/MnO_2_/PDA-Z_Her2_ NPs and X-ray irradiation exhibited the most profound inhibition of tumor growth, which was significantly more noticeable higher than that of the mice receiving MnO_2_-free Pt@mPDA/PDA-Z_Her2_ NPs + X-ray treatment (p < 0.001). These results indicated successful sensitization to RT induced by MnO_2_. No obvious body weight changes were observed during any of the treatments for the duration of the experiment (Fig. 8e).

**Fig. 9:**
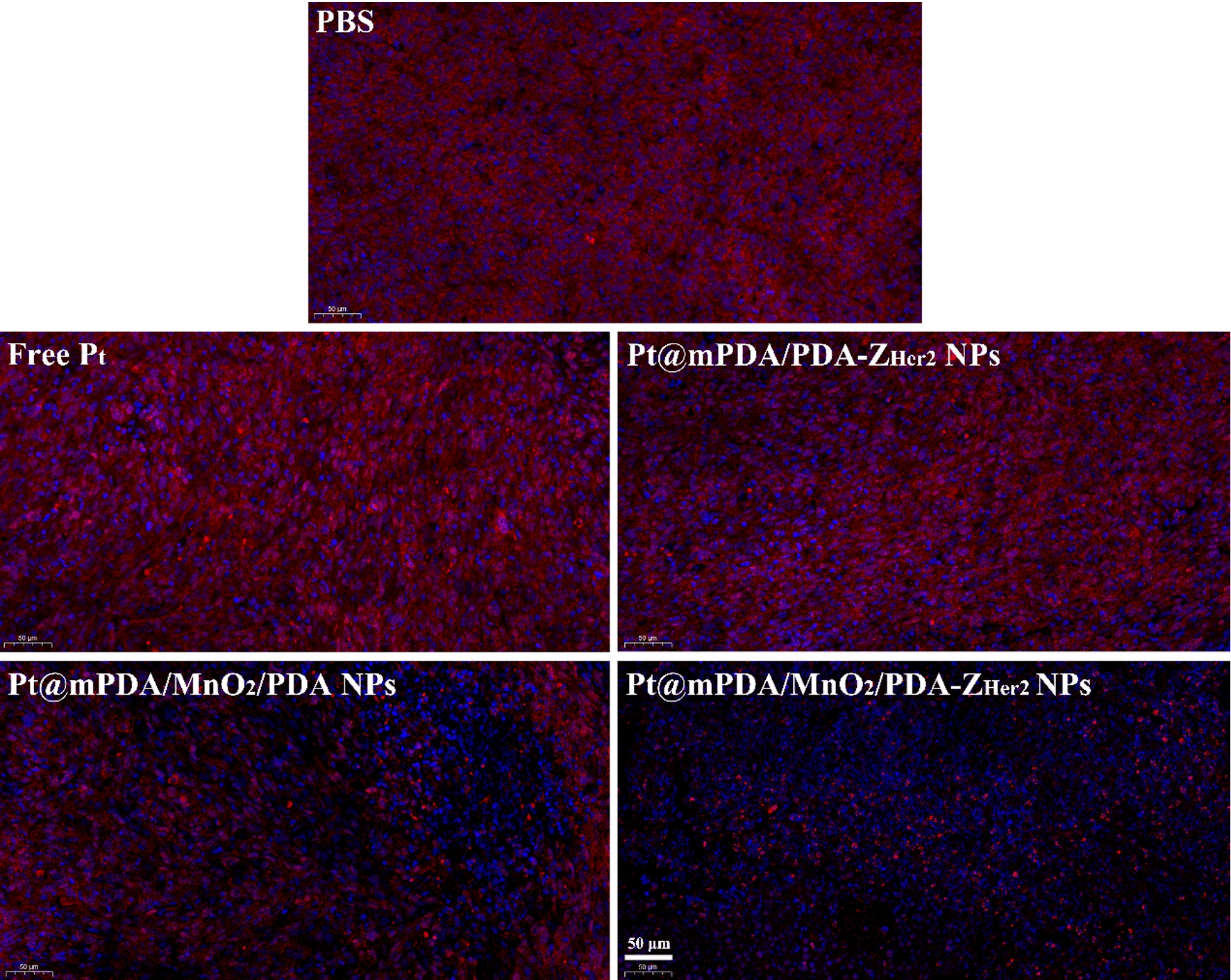
Immunofluorescence images of HIF-1α (red)-stained tumor slices excised from the various treatment groups. The nuclei of cells were visualized using DAPI (blue)

To better understand the mechanisms by which MnO_2_ sensitizes cells to RT, the HIF-1α expression level was measured in tumor tissue extracted from each treatment group by an immunofluorescence assay. Compared with that in all MnO_2_-free formulation treatment groups, the tumor tissues from the mice treated with Pt@mPDA/MnO_2_/PDA NPs showed a remarkable reduction in red fluorescence signal (HIF-1α) (Fig. 9). It indicated that TME in these mice was less hypoxic, which can be attributed to the decomposition of endogenous H_2_O_2_ to O_2_ by MnO_2_. Furthermore, with the targeted guiding of Z_Her2_ affibody, HIF-1α expression was further reduced by treatment with Pt@mPDA/MnO_2_/PDA-Z_Her2_ NPs, confirming that the tumor-targeting ability of the Z_Her2_ affibody contributed to the sensitization to RT.

### Safety evaluation

Evaluation of off-target toxicity is a prerequisite for in vivo use or clinical translation of novel therapeutic agents. Thus, the biocompatibility and biosafety of Pt@mPDA/MnO_2_/PDA-Z_Her2_ NPs were systematically evaluated by histology and serum biochemistry assays. H&E-stained images of major organs from all treatment mice showed no obvious tissue damage, compared with that from the PBS group (Additional file 1: Fig. S11), suggesting that the drug, materials, or both combined did not cause any significant systemic toxicity. Similarly, the levels of serum biomarkers (ALT, AST, UREA, CREA, and UA) have no significant difference from those in the control group (Additional file 1: Fig. S12), possibly due to the relatively low side effects of clinical cisplatin and biomimetic PDA, as well as the short treatment period. Therefore, this biocompatible nano-theranostic agent exhibits promising potential for clinical translation.

Her2 is overexpressed in a variety of human cancers and is closely related to cell proliferation, differentiation, adhesion, migration, and anti-apoptosis, as well as poor prognosis and rapid recurrence of tumors [48]. Antibody–drug conjugates (ADCs, such as FDA-approved trastuzumab, pertuzumab, and T-DM1), which integrate Her2-specific targeting antibodies with highly cytotoxic small-molecule chemotherapeutic agents, possess the ability to selectively deliver highly potent cytotoxic drugs to tumor sites and have become a powerful cancer-targeting treatment approach. However, these ADCs can have a poor response in the TME and do not offer any diagnostic abilities, as well as being high cost, which limits their wider usage. In the present study, a Her2-targeting Z_Her2_ affibody was generated using a genetic engineering approach and successfully coupled to Pt@mPDA/MnO_2_/PDA NPs. Compared to the widely used intact antibody, this engineered affibody has the advantage of higher specific affinity, stronger tissue penetrability, higher synthetic yield at a lower price, and low immunogenicity [48, 50]. Such affibodies can be employed to guide nano-agents for enhanced therapeutic efficacy. In addition, in marked contrast to monofunctional antibody–drug conjugates, the Z_Her2_ affibody with a nano-agent, which features specific targeting, RT sensitization, and diagnostic abilities, has been prepared and provide a new concept for clinical translation in tumor theranostics.

## Conclusion

In the present study, Her2-targeted cisplatin-loaded mPDA/MnO_2_/PDA NPs for MR imaging and enhanced chemo-radiotherapy of hypoxic tumors is reported. These NPs are biodegradable under a simulated tumor microenvironment, resulting in accelerated cisplatin release as well as O_2_ production by triggering decomposition of H_2_O_2_. Cell uptake experiments demonstrated that Z_Her2_ endowed the NPs with the ability to bind to Her2, achieving enhanced internalization of NPs into Her2-positive SKOV-3 cells. In vivo MRI studies revealed an obvious T_1_ contrast enhancement at the tumor site of mice receiving the NPs. Immunofluorescence assays showed that the presence of MnO_2_ significantly reduced the expression of HIF-1α and that the Cy5.5 fluorescently-labelled carrier has a high affinity for Her2. Chemotherapy results verified that Pt@mPDA/MnO_2_/PDA-Z_Her2_ NPs have a strong targeted antitumor efficacy. Combined with X-ray irradiation, the Pt@mPDA/MnO_2_/PDA-Z_Her2_ NPs can relieve tumor hypoxia and exhibit the superb inhibition of tumor growth. Hence, this multifunctional nanoplatform shows promising potential for clinical translation in tumor theranostics.

## Materials and methods

### Materials

Pluronic F127 (Mw: 12.6 kDa) was obtained from Sigma-Aldrich (USA). Dopamine hydrochloride (DA·HCl), 1, 3, 5-trimethylbenzene (TMB), dimethyl sulfoxide (DMSO), cisplatin (Pt, 65%), and cyanine-5.5 (Cy5.5) were purchased from Aladdin (Shanghai, China). Ammonium hydroxide (25.0–28.0%), ethanol, potassium permanganate (KMnO_4_), and hydrogen peroxide (H_2_O_2_, 30% v/v) were procured from Sinopharm Chemical Reagent Co., Ltd (Shanghai, China). Isopropyl-β-d-thiogalactoside (IPTG), 2-mercaptoethanol (2-ME), 6-carboxyfluorescence (6-FAM), 3-(4,5-dimethylthiazol-2-yl)-2,5-diphenyltetrazoliumbromide (MTT), glutathione (GSH), 2,7-dichlorodi-hydrofluorescein diacetate (DCFH-DA), calcein-AM, and propidium iodide (PI) were sourced from Sigma-Aldrich (USA). High-glucose Dulbecco’s modified Eagle’s medium (DMEM), foetal bovine serum, penicillin and streptomycin, trypsin-ethylenediaminetetraacetic acid (trypsin–EDTA), and phosphate-buffered saline (PBS) were obtained from Gibco (Carlsbad, CA). Tris (hydroxymethyl) aminomethane buffer (Tris-buffer, pH 8.6) and McCoy’s 5A medium supplemented with penicillin (100 U/mL) and streptomycin (100 U/mL) were acquired from Jiangsu KeyGEN BioTECH Co., Ltd (Nanjing, China). Human umbilical vein endothelial cells (HUVECs), breast cancer cell line MCF-7, and Her2-positive human ovarian cancer cell line SKOV-3 were provided by the Type Culture Collection of the Chinese Academy of Sciences (Shanghai, China). Deionized (DI) water (> 18.2 MΩ·cm) used for all experiments was purified using a Millipore system, and all chemicals were used without further purification.

### Preparation of Pt@mPDA/MnO_2_/PDA NPs

Mesoporous polydopamine nanoparticles (mPDA NPs) were synthesized according to a nano-emulsion assembly approach [51] with slight modifications. Pluronic F127 (2.0 g) and DA·HCl (1.0 g) were dissolved in 50% v/v ethanol (200 mL) and stirred at 1000 rpm for 3 h. Then, TMB (1 mL) was added dropwise to the mixture and sonicated for 5 min to allow for generation of the nano-emulsion. After stirring for another 30 min at 500 rpm, NH_4_OH (10 mL) was added to the resultant mixture while stirring under aerobic conditions to induce the self-polymerization of dopamine. After another 3 h of continuous reaction and then centrifugation, the mPDA NPs were collected and washed thoroughly with absolute ethanol and then dispersed in PBS for further use. For drug loading, DMSO (200 μL) containing cisplatin (25 mg) was added to the mPDA NPs (50 mg in 50 mL of PBS), followed by sonication for 30 min, and stirring for 24 h in the dark. The cisplatin-loaded mPDA NPs (denoted Pt@mPDA NPs) were collected after centrifugation and washed with DI water.

The Pt@mPDA NPs were re-dispersed into DI water (50 mL, pH 7.4), and KMnO_4_ (50 mg) was added while stirring at 400 rpm for 6 h. After sonication for another 6 h, the mixture was centrifuged and washed thoroughly with DI water, yielding Pt@mPDA/MnO_2_ NPs. Finally, the Pt@mPDA/MnO_2_ NPs were re-dispersed in tris-buffer (100 mL, pH = 8.6), and DA (50 mg) was added. The mixture was stirred (400 rpm) for 4 h, and the NPs were washed with DI water. This yielded Pt@mPDA/MnO_2_/PDA NPs, which were re-dispersed into DI water for further use.

### Preparation and characterization of the Z_Her2_ affibody

The Z_Her2_ affibody was expressed and purified according to our previous work [52], with some modifications. In detail, an artificial and optimized gene (5′-GCGGAGGCGAAGTACGCGAAAGAAATGCGTAACGCGTATTGGGAGATCGCGCTGCTGCCGAACCTGACCAACCAGCAAAAGCGTGCGTTCATTCGTAAACTGTACGACGATCCGAGCCAGAGCAGCGAGCTGCTGAGCGAAGCGAAGAAACTGAACGACAGCCAAGCGCCGAAGTGC-3′) encoding Z_Her2_ affibody molecule with an added cysteine at the C-terminus of protein was synthesized by GenScript (Nanjing, China) and cloned into pQE30 at the *BamHI* and *SalI* sites to construct the expression plasmid pQE30-Z_Her2_. The plasmid was transformed into *E. coli* M15 and induced overnight with 0.05 mM IPTG at 28 °C. Subsequently, the cells were collected by centrifugation, resuspended in lysis buffer (50 mM phosphate, pH 8.0, 300 mM NaCl, and 20 mM imidazole) and sonicated on ice for 30 min to lyse the cells. The recombinant protein in the supernatant was purified using Ni–NTA affinity chromatography according to the manufacturer’s instructions (GenScript, Nanjing, China). The fractions collected during the Z_Her2_ preparation process were detected using sodium dodecyl sulphate polyacrylamide gel electrophoresis (SDS-PAGE) and analyzed using Image J software (Bethesda, MD, USA). The purified affibody was dialyzed against phosphate-buffered saline (PBS, 137 mM NaCl, 2.7 mM KCl, 4.3 mM Na_2_HPO_4_, and 1.4 mM KH_2_PO_4_, pH 7.4) and then quantified using a Bradford Protein Assay Kit (Beyotime, Jiangsu, China).

The aggregation form of Z_Her2_ was detected using SDS-PAGE with or without 2-mercaptoethanol (2-ME) in the loading buffer. For the specific cell binding assay, Z_Her2_ was labelled with 6-FAM (Ruixi Biology, Xi’an, China) according to the manufacturer’s protocol. In brief, 6-FAM (10 μL; 10 mg/mL in DMF) was added to Z_Her2_ solution (5 mL; 2 mg/mL; pH 8.3 in PBS). The reaction was allowed to proceed for 1 h at 25 °C in the dark before the mixture was dialyzed against PBS (pH 7.4) at 4 °C for 48 h to remove unreacted 6-FAM. The conjugation of 6-FAM to the Z_Her2_ affibody was verified by SDS-PAGE.

### Conjugation of Z_Her2_ affibody to NPs

The Pt@mPDA/MnO_2_/PDA-Z_Her2_ NPs were constructed by conjugating Z_Her2_ to Pt@mPDA/MnO_2_/PDA NPs via a Michael addition/Schiff base reaction between the amino group and the oxidized quinone form of catechol groups under weak alkaline pH conditions [53, 54]. The Pt@mPDA/MnO_2_/PDA NPs were dispersed in tris-buffer (100 mL, 0.5 mg/mL), and Z_Her2_ solution (1 mL; 1 mg/mL in PBS) was added, followed by sonication (40 kHz, 70 W) for 30 min. The mixture was then stirred overnight at 25 °C. The unreacted Z_Her2_ was removed by centrifugation, and the NPs were thoroughly washed with DI water. This yielded Pt@mPDA/MnO_2_/PDA-Z_Her2_ NPs, which was re-dispersed in PBS for further use.

### Characterization of NPs

The morphology of the NPs was observed by transmission electron microscopy (TEM, JEOL 2010F) at an accelerated voltage of 200 kV. The surface area and pore size of the mPDA NPs were measured using an automated surface area and porosity analyzer (Quantachrome, Autosorb-iQ). X-ray photoelectron spectroscopy (XPS) was performed using a Thermo Fisher ESCALAB 250Xi spectrometer to determine the chemical state of MnO_2_. Zeta potential and particle size distribution were measured using a Malvern Zetasizer (Nano-ZS, Malvern, UK). UV–Vis absorbance spectra were recorded on a UV-2100 spectrophotometer. The concentration of MnO_2_, cisplatin, and Z_Her2_ in the NPs were analyzed by measuring Mn, Pt, and S elements using inductively coupled plasma-atomic emission spectrometry (ICP-AES, Prodigy, LEEMAN).

### Biodegradation and in vitro drug release

Pt@mPDA/MnO_2_/PDA NPs were dispersed in PBS (50 mL; pH 7.4) and incubated with different concentration of either (1) H_2_O_2_ (1 mM); (2) GSH (2 mM); (3) H_2_O_2_ (1 mM) and GSH (2 mM); (4) H_2_O_2_ (1 mM), GSH (5 mM) at 37 °C with shaking at 120 rpm for 14 d. At each specified time point, a sample (1 mL) was removed for UV–Vis absorbance measurement. After 2 weeks, each sample was collected and the extent of degradation was determined using TEM.

The concentration of drug released from the NPs was measured according to the dialysis method described previously [14], with some modifications. In brief, Pt@mPDA/MnO_2_/PDA NPs (3 mg) were dispersed in PBS (2 mL, pH 7.4) and then loaded into a dialysis bag (MWCO = 7000 Da) and immersed in PBS (18 mL; pH 7.4 and 5.5) containing different concentration of H_2_O_2_ (0 or 1 mM) and GSH (0 or 5 mM). All samples were incubated at 37 °C with shaking (120 rpm) for 2 days. At predetermined time points, some external medium (1 mL) was removed, and the samples were supplemented with an equal volume of fresh pre-heated medium. The free cisplatin release profile from dialysis bag was measured using the same method. The concentration of the released cisplatin was determined quantitatively by ICP-AES. All experiments were performed in triplicate.

### Measurement of dissolved O_2_ and detection of ROS in vitro

The ability of MnO_2_-based NPs to catalyze the decomposition of H_2_O_2_ to O_2_ was measured using a dissolved oxygen meter (JPSJ-605, INESA). Pt@mPDA/MnO_2_/PDA NPs were dispersed in PBS (50 mL; pH 7.4 and 5.5; [MnO_2_] = 2 μg/mL) and then transferred into a double-neck flask. H_2_O_2_ (30% w/v) was added at a final concentration of 1 mM. Blank PBS (pH 7.4) or PBS (pH 7.4) containing H_2_O_2_ was used as the control medium. The concentration of dissolved O_2_ was measured at predetermined time points.

The RT-sensitizing effect of MnO_2_-containing NPs caused by catalyzing the decomposition of H_2_O_2_ to O_2_ was measured using a methylene blue (MB) degradation method. MB solution (50 mL; 10 μg/mL) containing H_2_O_2_ (8 mM) was separately treated with (1) X-Ray (6 Gy), (2) Pt@mPDA/PDA-Z_Her2_ NPs (5 mg) + X-Ray (6 Gy), (3) Pt@mPDA/MnO_2_/PDA-Z_Her2_ (5 mg), (4) Pt@mPDA/MnO_2_/PDA-Z_Her2_ (5 mg) + X-Ray (6 Gy). After incubation for 30 min at 37 ℃, the MB absorbance of each sample was measured.

### GSH-activated T_1_-weighted MRI

Pt@mPDA/MnO_2_/PDA NPs at different concentrations of Mn ([Mn] = 1 mM, 2 mM, 3 mM, 4 mM, and 5 mM in PBS) were each treated with or without 2 mM GSH. After 20 min, the T_1_-weighted relaxation times were measured using a 0.5 T NMI20 NMR Analyzing and Imaging system (Niumag, Shanghai, China) at 25 °C. The test parameters were set according to a previous publication [14]. T_1_ relaxivity (r_1_) was acquired by fitting a linear function through 1/T_1_ as a function of Mn concentration. In addition, T_1_ MRI was performed for samples at different concentrations using a clinical MR system (1.5 T, Siemens Magnetom Symphony).

### In vitro cellular uptake evaluation

The expression of Her2 in the breast cancer cell line MCF-7 and the human ovarian cancer cell line SKOV-3 was evaluated. MCF-7 and SKOV-3 cells were incubated in DMEM or McCoy’s 5A medium supplemented with 1% v/v penicillin, 1% v/v streptomycin, and 10% v/v foetal bovine serum. The cells were cultured at 37 °C in a 5% CO_2_ humidified atmosphere. MCF-7 and SKOV-3 cells (2 × 10^5^) were digested and resuspended in DMEM or McCoy’s 5A medium containing 2 or 4 μg/mL FITC-anti-Her2 antibody (Sino Biological, Beijing, China) at 37 °C for 2 h. After incubation, the cells were washed three times with PBS and resuspended in PBS (0.5 mL). The FITC fluorescence intensity was determined using a Becton–Dickinson FACScan analyzer (Frankin, CA, USA). Three independent experiments were conducted. Visualization of the distribution of FITC-anti-Her2 antibody in cells was further analyzed by confocal laser scanning microscopy (CLSM, Carl Zeiss LSM 700). Specifically, MCF-7 or SKOV-3 cells were seeded into 24-well plates and cultured at 37 °C. After 12 h, the cells were incubated with 2 or 4 μg/mL FITC-anti-Her2 antibody for a further 2 h. The cells were washed three times with PBS, fixed with 4% paraformaldehyde for 15 min at 4 °C, and then washed again three times with PBS. Finally, the cells were immediately observed using CLSM.

The specific affinity between Z_Her2_ and Her2-positive cancer cell lines was evaluated. Her2-negative MCF-7 cells or Her2-positive SKOV-3 cells were seeded in a confocal dish (5 × 10^4^ cells per dish) and incubated for 12 h. Thereafter, the medium was aspirated and replaced with fresh medium containing FAM-Z_Her2_ (2 mL; 50 μg/mL). After another 2 h of incubation, the cells were washed three times with PBS and then fixed with 4% paraformaldehyde for 15 min at 4 °C. The cell nuclei were stained with DAPI (1 mL, 10 μg/mL) for 5 min and then washed three times with PBS. Finally, the cells were immediately observed using CLSM. The affinity between FAM-Z_Her2_ and SKOV-3 cells was also evaluated by flow cytometry (FCM). Approximately 2 × 10^5^ digested cells were incubated with FAM-Z_Her2_ (50 μg/mL) at 37 °C for 2 h. For Her2 receptor blocking experiments, SKOV-3 cells were pre-incubated with Z_Her2_ (20 μg/mL) for 1 h and then incubated with FAM-Z_Her2_ (50 μg/mL) for another 2 h. After that, the cells were washed three times with PBS and re-dispersed in PBS (0.5 mL) for FCM assay.

FCM was used to evaluate the affinity of monomeric and dimeric affibodies to Her2 receptors. Briefly, SKOV-3 cells (2 × 10^5^) were digested and incubated with the same molar concentration (100 nM) of monomeric (in the presence of 2-ME) or dimeric (in the absence of 2-ME) FAM-Her2 affibody at 37 °C for 2 h. After incubation, the cells were washed three times with PBS and redispersed in PBS (0.5 mL). The FAM fluorescence intensity was determined by FCM.

To visually track the distribution of the NPs in cells, Cy5.5-loaded NPs (Cy5.5@mPDA/MnO_2_/PDA or Cy5.5@mPDA/MnO_2_/PDA-Z_Her2_ NPs) were prepared following the same method as described above. The SKOV-3 cellular uptake of these NPs was evaluated following a protocol similar to that described above, except that an FBS-free medium containing Cy5.5@mPDA/MnO_2_/PDA or Cy5.5@mPDA/MnO_2_/PDA-Z_Her2_ NPs (50 μg/mL; in PBS) was added after the initial culture. To investigate Her2-dependent binding, SKOV-3 cells were pre-incubated with free Z_Her2_ (20 μg/mL) for 1 h prior to incubation with Cy5.5@mPDA/MnO_2_/PDA-Z_Her2_ NPs (50 μg/mL). Finally, the cells were treated and probed by CLSM as described above.

FCM was performed to semi-quantify the uptake of these two NPs by SKOV-3 cells. Approximately 2 × 10^5^ cells were incubated with Cy5.5@mPDA/MnO_2_/PDA or Cy5.5@mPDA/MnO_2_/PDA-Z_Her2_ NPs (50 μg/mL; in PBS) after a 1 h pre-treatment with or without free Z_Her2_ (20 μg/mL). After 4 h incubation at 37 °C, the cells were washed three times with PBS and re-dispersed in 0.5 mL PBS for FCM analysis.

### Cytotoxicity assays

The cytocompatibility of drug-free mPDA/MnO_2_/PDA-Z_Her2_ NPs to HUVECs was first determined by performing the MTT assay. HUVECs (~ 1 × 10^4^) were seeded into 96-well plates and cultured overnight at 37 °C in a 5% CO_2_ humidified environment. The medium was replaced with fresh DMEM (200 μL) containing different concentrations of mPDA/MnO_2_/PDA-Z_Her2_ NPs (1, 5, 10, 20, 50, 100, and 250 μg/mL), and the cells were incubated for another 24 h. Subsequently, the medium of each well was carefully discarded followed by the addition of MTT solution (20 μL, 10 μg/mL), and the cells were incubated for an additional 4 h. Finally, DMSO (200 μL) was added after removing the medium, and the absorbance of the wells at 570 nm was measured with a microplate reader (Multiskan FC, Thermo Scientific).

The in vitro anticancer efficacy of different cisplatin formulations was investigated using a method similar to that described above, except that medium (200 μL) containing free cisplatin, Pt@mPDA/MnO_2_/PDA NPs, or Pt@mPDA/MnO_2_/PDA-Z_Her2_ NPs ([cisplatin] = 0.6, 3, 6, 12, 24, or 48 μg/mL) was added after the initial incubation. Data are reported as mean ± S.D., with three independently performed experiments each containing three replicates. The cells treated with free cisplatin, Pt@mPDA/MnO_2_/PDA NPs, or Pt@mPDA/MnO_2_/PDA-Z_Her2_ NPs ([cisplatin] = 48 μg/mL) for 24 h were further stained with PI (staining dead cells red) and calcein-AM (staining live cells green) in PBS solution for 30 min at 37 °C in the dark and then imaged by inverted fluorescence microscopy (Carl Zeiss). For all experiments, PBS-treated cells were used as the control.

### Measurement of intracellular ROS levels

The levels of intracellular ROS were measured using DCFH-DA to evaluate the RT-sensitizing effect of MnO_2_. SKOV-3 cells were seeded onto 24-well plates at 50,000 cells/well and maintained in McCoy’s 5A medium. After 12 h incubation at 37 °C, cells were treated with either (1) PBS, (2) PBS + X-ray, (3) MnO_2_-free mPDA/PDA-Z_Her2_ NPs (50 μg/mL; in PBS) + X-ray, (4) mPDA/MnO_2_/PDA-Z_Her2_ NPs (50 μg/mL; in PBS), and (5) mPDA/MnO_2_/PDA-Z_Her2_ NPs (50 μg/mL; in PBS) + X-ray. In samples receiving X-ray treatment, the cells were irradiated with X-rays (6 Gy) after 4 h of incubation. After an additional 1 h of incubation, DCFH-DA solution (1 mL; 10 μM) was added to the cells for another 30 min. Subsequently, the fluorescence images were acquired with inverted fluorescence microscopy (Carl Zeiss).

### Hemocompatibility assay

Fresh blood was obtained from female Sprague–Dawley (SD) rats and centrifuged at 1500 rpm for 15 min to separate red blood cells (RBCs). RBCs were then treated with a suspension of Pt@mPDA/MnO_2_/PDA NPs or Pt@mPDA/MnO_2_/PDA-Z_Her2_ NPs in PBS (5 mL, 250 μg/mL). PBS (5 mL; pH 7.4) and Triton X-100 (5 mL; 1% w/v) were used as negative controls and positive controls, respectively. Each sample was incubated at 37 ℃ for 1 h, and the intact RBCs were separated by centrifugation (1500 rpm; 15 min). The supernatant from each tube was collected and the amount of hemoglobin release was determined by reading the absorbance at 540 nm in a microplate reader (Multiskan FC, Thermo Scientific). The formula below was used to calculate the percentage of RBC hemolysis and each experiment was conducted in triplicate.

$${\text{Hemolysis }}\% \, = \, \left( {{\text{OD }}\left[ {{\text{test}}} \right] \, {-}{\text{ OD }}\left[ {\text{negative control}} \right]} \right) \times { 1}00/{\text{OD }}\left[ {\text{positive control}} \right]$$

### Animals and tumor model

All animal experiments were carried out with full authorization by the ethics committee for animal care of Shandong Cancer Hospital and Institute Shandong, First Medical University and Shandong Academy of Medical Sciences (Approval No. SDTHEC2020004083). Female BALB/C nude mice (SPF grade, 4–6 weeks old) were acquired from Beijing Huafukang Bioscience Co. Inc. (Beijing, China). Tumors were established by subcutaneous injection of SKOV-3 cells (1 × 10^6^) dispersed in PBS (100 μL) into the right flank of each mouse. The tumor volume was monitored in real-time and calculated as length × width^2^/2.

### In vivo MRI and biodistribution

In order to demonstrate the TME-triggered MRI ability of the MnO_2_ layer, PBS (50 μL) containing Pt@mPDA/MnO_2_/PDA NPs ([Mn] = 50 mM) was indirectly injected into the tumor sites or the muscle on the opposite side to the tumors. At pre-determined time points (0 min, 5 min, and 120 min), the SKOV-3 tumor-bearing mice were scanned using an MR analysis and imaging system (1.5 T, Siemens Magnetom Symphony).

When the tumor volume reached approximately 200 mm^3^, Pt@mPDA/MnO_2_/PDA NPs or Pt@mPDA/MnO_2_/PDA-Z_Her2_ NPs ([Mn] = 3 mM, 100 μL in PBS per mouse) were intravenously injected into the tumor-bearing mice, and T_1_-weighted MR images were obtained at different time points after injection. The following MR scanning parameters were used: TE = 16.9 ms, TR = 760 ms, FOV = 10 cm × 10 cm, slice thickness = 3 mm, and point resolution = 512 mm × 512 mm.

The mice were sacrificed at 12 h after receiving NPs, and the heart, liver, spleen, lung, kidney, brain, muscle, and tumors were extracted and weighed and then dissolved in aqua regia solution (2 mL, 65 °C) for 24 h. The mice intravenously injected with free cisplatin (200 μL; 200 μg/mL in PBS) were used as control group. Finally, the Pt content in each tissue and tumor was quantified by ICP-AES.

### Immunofluorescence and bio-TEM assays

The SKOV-3 tumor-bearing mice were intravenously injected with PBS containing Cy5.5@mPDA/MnO_2_/PDA NPs or Cy5.5@mPDA/MnO_2_/PDA-Z_Her2_ NPs (100 μL; 1 mg/mL). For the immunofluorescence assay, tumors were collected at 12 h post-injection and fixed with OCT (Sakura) for cryo-sectioning. The tumor sections were incubated overnight with a rabbit anti-mouse Her2 primary antibody (dilution 1:200, Abcam) at 4 °C and then for 60 min with a goat anti-rabbit secondary antibody (dilution 1:200, Abcam) at 37 °C. The cell nuclei were stained with DAPI. Finally, the obtained slices were scanned using an imaging system (Nikon DS-U3).

For bio-TEM, tumors were treated with 1% OsO_4_ for 2 h at 25 °C, then embedded in resin after the cells were dehydrated. Ultrathin sections(70–90 nm) of tumor tissue were cut and analyzed by bio-TEM (Hitachi HT7700, Tokyo, Japan).

### In vivo antitumor efficacy and safety evaluation

When the volume of tumors reached ~ 50 mm^3^, the tumor-bearing mice were randomly divided into four groups (n = 5) and intravenously injected with either (1) PBS, (2) free cisplatin in PBS, (3) Pt@mPDA/MnO_2_/PDA NPs in PBS, or (4) Pt@mPDA/MnO_2_/PDA-Z_Her2_ NPs in PBS (200 μL; dose of cisplatin = 2 mg/kg) every 2 days. Tumor volume and body weights were monitored and recorded every day after the first injection. On the 14^th^ day, 0.5 mL of blood from each group of mice (n = 3) was withdrawn, after which the experiment was halted. The tumors were collected and stained with terminal deoxynucleotidyl transferase UTP nick end labelling (TUNEL) for the apoptosis assay, while the major organs (heart, liver, spleen, lung, and kidney) were extracted and stained with haematoxylin and eosin (H&E) for histological analysis.

For chemo-radiation combined therapy, when the volume of tumors reached ~ 50 mm^3^, four groups of tumor-bearing mice (n = 5) were treated with either (5) PBS (200 μL), (6) PBS (200 μL) + X-ray, (7) MnO_2_-free Pt@mPDA/PDA-Z_Her2_ NPs in PBS (200 μL) + X-ray, or (8) Pt@mPDA/MnO_2_/PDA-Z_Her2_ NPs in PBS (200 μL) + X-ray. All groups of mice except for group (5) received cisplatin and MnO_2_ doses of 2 mg/kg and 3.4 mg/kg, respectively, every 2 days. For radiotherapy, mice received X-ray radiation at a dose of 6 Gy for 24 h post-injection every 4 days. Tumor sizes and body weights were recorded every day. After 14 days the mice were sacrificed, and tumors were collected for volume measurement and weighing. Finally, to evaluate tumor hypoxia levels, the tumor tissues from groups (1)–(4) and group (7) were HIF-1α stained according to the manufacturer’s instructions.

### Statistical analysis

All results are presented as mean ± standard deviation (S.D.), and between-group comparisons were evaluated using one-way ANOVA. A p value < 0.05 indicates statistical significance, and data are represented as (*) for p < 0.05, (**) for p < 0.01, and (***) for p < 0.001. NS stands for not statistically significant.

## Supplementary Information

**Additional file 1: Fig. S1.** (a) N_2_ adsorption/desorption isotherms of mPDA NPs and (b) the corresponding pore-size distribution curves. (c) Zeta potential values of cisplatin, mPDA, Pt@mPDA, Pt@mPDA/MnO_2_ and Pt@mPDA/MnO_2_/PDA NPs dispersed in water (pH 7.4). **Fig. S2.** (a) XPS full survey spectra and (b) Mn 2p XPS spectra of mPDA/MnO_2_/PDA NPs. **Fig. S3.** UV–Vis absorption spectra of Pt@mPDA/MnO_2_/PDA NPs in (a) blank PBS (pH 7.4) and PBS (pH 7.4) containing (b) 1 mM H_2_O_2_, (c) 2 mM GSH, (d) 1 mM H_2_O_2_ + 2 mM GSH, and (e) 1 mM H_2_O_2_ + 5 mM GSH at different time points; (f) the absorption spectra comparison of aqueous dispersions of NPs after 14 d under different conditions. **Fig. S4**. UV–Vis absorption spectra of MB solution after treating with X-ray (6 Gy), Pt@mPDA/PDA NPs + X-ray (6 Gy), Pt@mPDA/MnO_2_/PDA NPs, and Pt@mPDA/MnO_2_/PDA NPs + X-ray (6 Gy) for 30 min. Blank MB solution was used as control. **Fig. S5.** (a) DLS sizes of Pt@mPDA/MnO_2_/PDA and Pt@mPDA/MnO_2_/PDA-Z_Her2_ NPs in water. (b) DLS sizes of Pt@mPDA/MnO_2_/PDA and Pt@mPDA/MnO_2_/PDA-Z_Her2_ NPs in PBS over 24 h; **Fig. S6.** FCM data for (a) MCF-7 or (b) SKOV-3 cells after treatment with PBS, 2 or 4 μg/mL of FITC-labelled anti-Her2 antibody for 2 h and showing the corresponding quantified fluorescence intensity. (c) Fluorescence images for MCF-7 or SKOV-3 cells after treatment with PBS and 2 or 4 μg/mL of FITC-labelled anti-Her2 antibody for 2 h. **Fig. S7.** (a) CLSM images of MCF-7 and SKOV-3 cells after 2 h incubation with FAM-Z_Her2_. (b) Flow cytometry data for: untreated SKOV-3 cells; SKOV-3 cells incubated for 2 h with FAM-Z_Her2_ and cells pre-incubated with Z_Her2_ for 1 h before being exposed to FAM-Z_Her2_ for 2 h, and corresponding quantified fluorescence intensity. **Fig. S8.** (a) Flow cytometry data for: untreated SKOV-3 cells; SKOV-3 cells incubated for monomeric (in the presence of 2-ME) or dimeric (in the absence of 2-ME) FAM-Her2 affibody at 37 °C for 2 h. (b) MTT viability results for HUVECs treated with different concentration of mPDA/MnO_2_/PDA-Z_Her2_ NPs. **Fig. S9.** (a) Microscope images of RBCs incubated with (II) PBS, (II) Triton-100, (III) Pt@mPDA/MnO_2_/PDA NPs, (IV) Pt@mPDA/MnO_2_/PDA-Z_Her2_ NPs and (b) corresponding pictures after centrifugation (5000 rpm, 10 min). (c) Hemocompatibility data. **Fig. S10.** Biodistribution of free cisplatin (Pt), Pt@mPDA/MnO_2_/PDA and Pt@mPDA/MnO_2_/PDA-Z_Her2_ NPs in mice bearing SKOV-3 tumor grafts. **Fig. S11.** Representative H&E-stained images of the major organs collected after in vivo chemotherapy experiment. **Fig. S12.** Blood biochemical analyses of the mice from chemotherapy experiment.

## Data Availability

All data generated or analyzed during this study are included in this published article.

## Abbreviations

RT: Radiotherapy
ROS: Reactive oxygen species
TME: Tumor microenvironment
HIF-1α: Hypoxia-inducible factor 1α
MRI: Magnetic resonance imaging
Her2: Human epidermal growth factor receptor 2
Pt: Cisplatin
mPDA: Mesoporous polydopamine
