# Supplementary material for: Dimeric Her2-specific affibody mediated cisplatin-loaded nanoparticles for tumor enhanced chemo-radiotherapy
Source: J Nanobiotechnology. 2021 May 13;19:138. doi: 10.1186/s12951-021-00885-6 (PMC8120847; doi:10.1186/s12951-021-00885-6)
Supplement: Supplementary file 1 — Additional file 1: Fig. S1. (a) N2 adsorption/desorption isotherms of mPDA NPs and (b) the corresponding pore-size distribution curves. (c) Zeta potential values of cisplatin, mPDA, Pt@mPDA, Pt@mPDA/MnO2 and Pt@mPDA/MnO2/PDA NPs dispersed in water (pH 7.4). Fig. S2. (a) XPS full survey spectra and (b) Mn 2p XPS spectra of mPDA/MnO2/PDA NPs. Fig. S3. UV–Vis absorption spectra of Pt@mPDA/MnO2/PDA NPs in (a) blank PBS (pH 7.4) and PBS (pH 7.4) containing (b) 1 mM H2O2, (c) 2 mM GSH, (d) 1 mM H2O2 + 2 mM GSH, and (e) 1 mM H2O2 + 5 mM GSH at different time points; (f) the absorption spectra comparison of aqueous dispersions of NPs after 14 d under different conditions. Fig. S4. UV–Vis absorption spectra of MB solution after treating with X-ray (6 Gy), Pt@mPDA/PDA NPs + X-ray (6 Gy), Pt@mPDA/MnO2/PDA NPs, and Pt@mPDA/MnO2/PDA NPs + X-ray (6 Gy) for 30 min. Blank MB solution was used as control. Fig. S5. (a) DLS sizes of Pt@mPDA/MnO2/PDA and Pt@mPDA/MnO2/PDA-ZHer2 NPs in water. (b) DLS sizes of Pt@mPDA/MnO2/PDA and Pt@mPDA/MnO2/PDA-ZHer2 NPs in PBS over 24 h; Fig. S6. FCM data for (a) MCF-7 or (b) SKOV-3 cells after treatment with PBS, 2 or 4 μg/mL of FITC-labelled anti-Her2 antibody for 2 h and showing the corresponding quantified fluorescence intensity. (c) Fluorescence images for MCF-7 or SKOV-3 cells after treatment with PBS and 2 or 4 μg/mL of FITC-labelled anti-Her2 antibody for 2 h. Fig. S7. (a) CLSM images of MCF-7 and SKOV-3 cells after 2 h incubation with FAM-ZHer2. (b) Flow cytometry data for: untreated SKOV-3 cells; SKOV-3 cells incubated for 2 h with FAM-ZHer2 and cells pre-incubated with ZHer2 for 1 h before being exposed to FAM-ZHer2 for 2 h, and corresponding quantified fluorescence intensity. Fig. S8. (a) Flow cytometry data for: untreated SKOV-3 cells; SKOV-3 cells incubated for monomeric (in the presence of 2-ME) or dimeric (in the absence of 2-ME) FAM-Her2 affibody at 37 °C for 2 h. (b) MTT viability results for HUVECs treated with different concentrati [file 12951_2021_885_MOESM1_ESM.docx]

**Dimeric Her2-specific affibody mediated cisplatin-loaded nanoparticles**

**for tumor enhanced chemo-radiotherapy**

Haijun Wang^1,4,#^, Dianlong Jia^3,#^, Dandan Yuan^5^, Xiaolei Yin^4^, Fengjiao Yuan^6^, Feifei Wang^3^, Wenna Shi^1^, Hui Li^1^, Li-Min Zhu^2*^, Qing Fan^1*^

^1^ Department of Pharmacy, Shandong Cancer Hospital and Institute, Shandong First Medical University and Shandong Academy of Medical Sciences, Jinan 250117, China

^2^ College of Chemistry, Chemical Engineering and Biotechnology, Donghua University, Shanghai 201620, China

^3^ Laboratory of Drug Discovery and Design, School of Pharmacy, Liaocheng University, Liaocheng 252000, China

^4^ School of Life Sciences, Shandong First Medical University and Shandong Academy of Medical Sciences, Taian 271016, China

^5^ Department of Digestive Oncology, Shandong Cancer Hospital and Institute, Shandong First Medical University and Shandong Academy of Medical Sciences, Jinan 250117, China

^6^ Joint Laboratory for Translational Medicine Research, Liaocheng People’s Hospital, Liaocheng 252000, China

*Corresponding authors: E-mail address: [lzhu@dhu.edu.cn](file:///C:\Users\User\Downloads\lzhu@dhu.edu.cn) (Li-Min Zhu); [qingfan0708@yahoo.com](file:///C:\Users\User\Downloads\qingfan0708@yahoo.com) (Qing Fan)

^#^ These authors contributed equally.


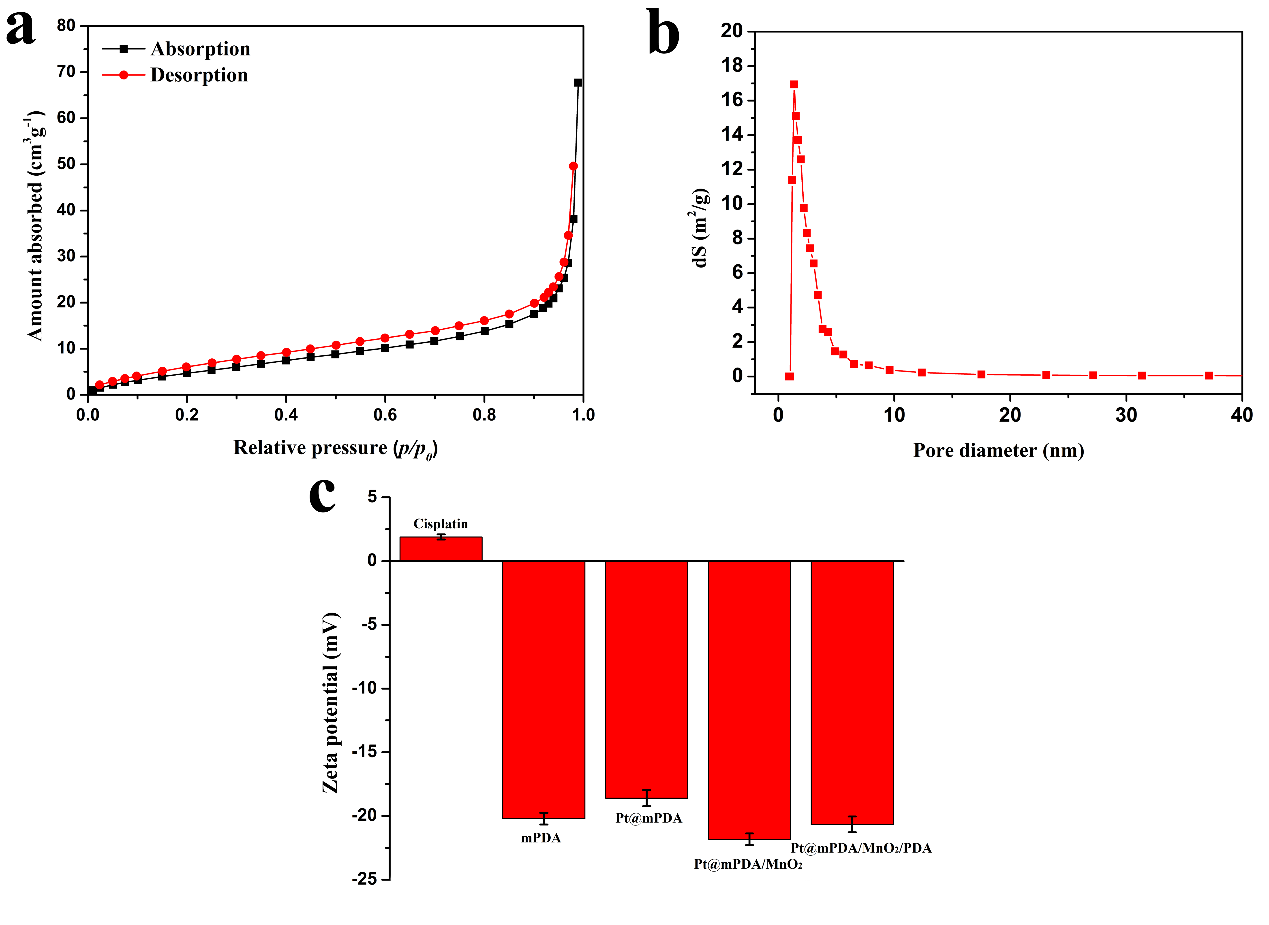


**Fig. S1.** (a) N_2_ adsorption/desorption isotherms of mPDA NPs and (b) the corresponding pore-size distribution curves. (c) Zeta potential values of cisplatin, mPDA, Pt@mPDA, Pt@mPDA/MnO_2_ and Pt@mPDA/MnO_2_/PDA NPs dispersed in water (pH 7.4).


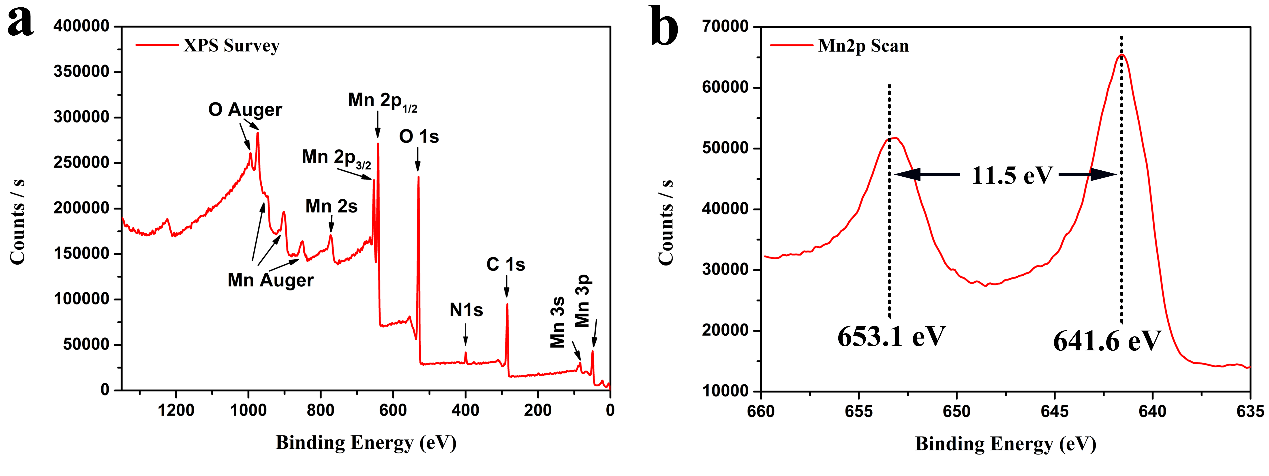


**Fig. S2.** (a) XPS full survey spectra and (b) Mn 2p XPS spectra of mPDA/MnO_2_/PDA NPs.


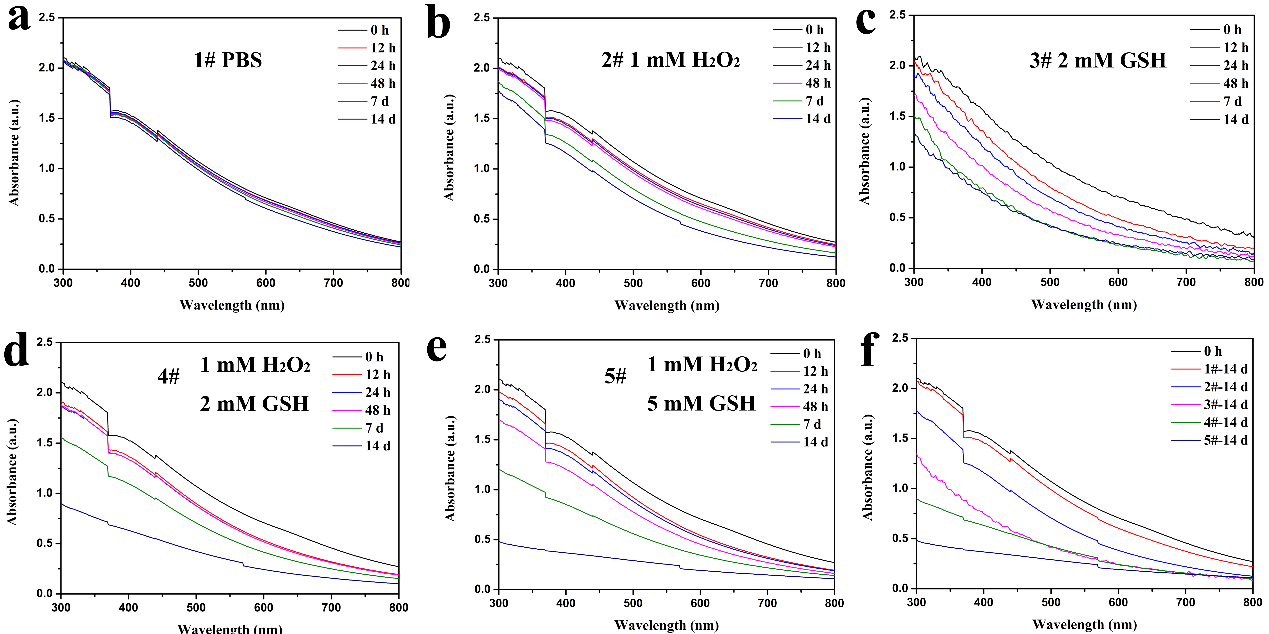


**Fig. S3.** UV-Vis absorption spectra of Pt@mPDA/MnO_2_/PDA NPs in (a) blank PBS (pH 7.4) and PBS (pH 7.4) containing (b) 1 mM H_2_O_2_, (c) 2 mM GSH, (d) 1 mM H_2_O_2_ + 2 mM GSH, and (e) 1 mM H_2_O_2_ + 5 mM GSH at different time points; (f) the absorption spectra comparison of aqueous dispersions of NPs after 14 d under different conditions.


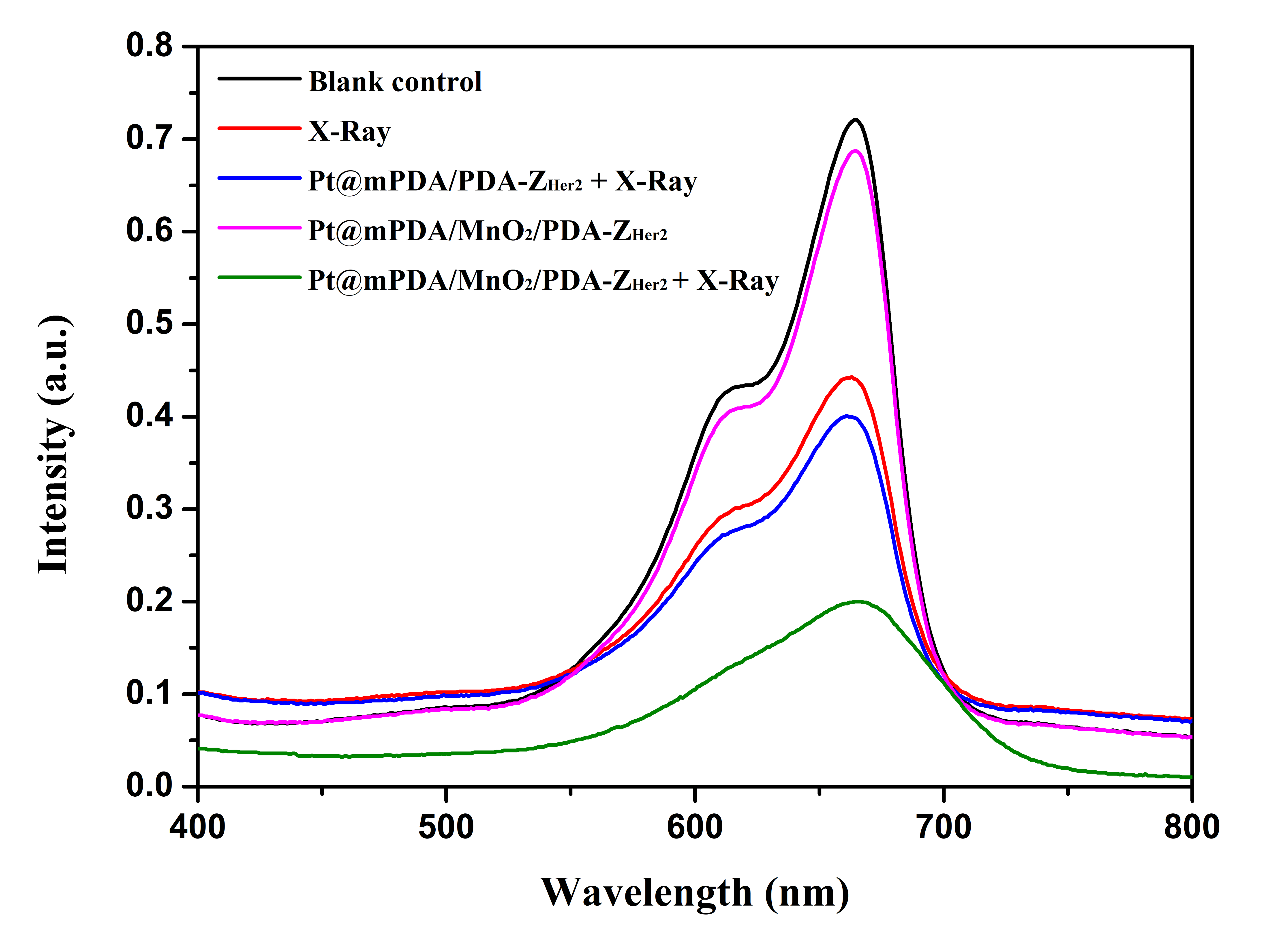


**Fig. S4**. UV-Vis absorption spectra of MB solution after treating with X-ray (6 Gy), Pt@mPDA/PDA NPs + X-ray (6 Gy), Pt@mPDA/MnO2/PDA NPs, and Pt@mPDA/MnO_2_/PDA NPs + X-ray (6 Gy) for 30 min. Blank MB solution was used as control.


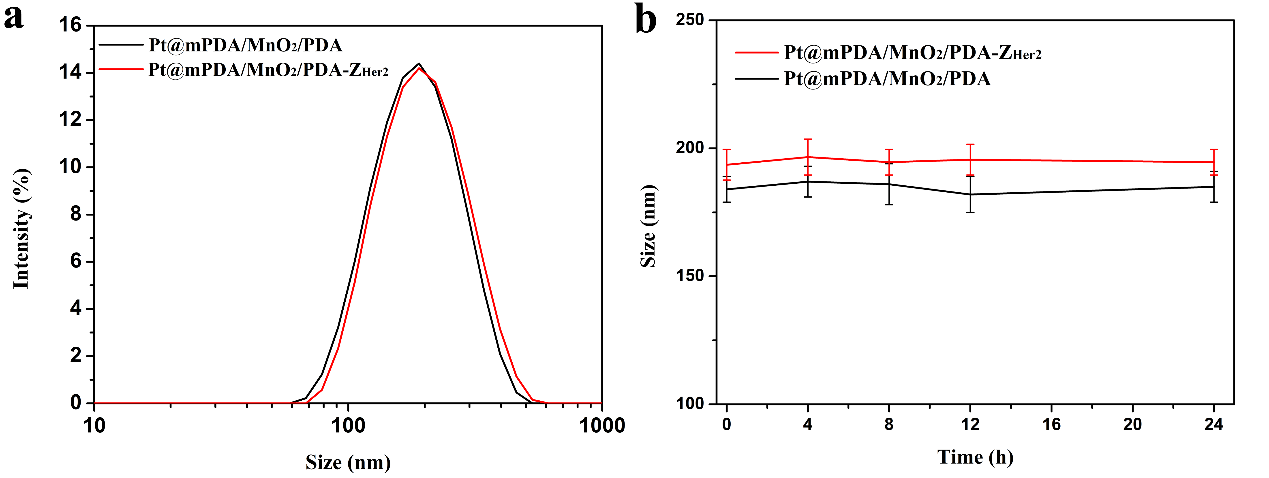


**Fig. S5.** (a) DLS sizes of Pt@mPDA/MnO_2_/PDA and Pt@mPDA/MnO_2_/PDA-Z_Her2_ NPs in water. (b) DLS sizes of Pt@mPDA/MnO_2_/PDA and Pt@mPDA/MnO_2_/PDA-Z_Her2_ NPs in PBS over 24 h;


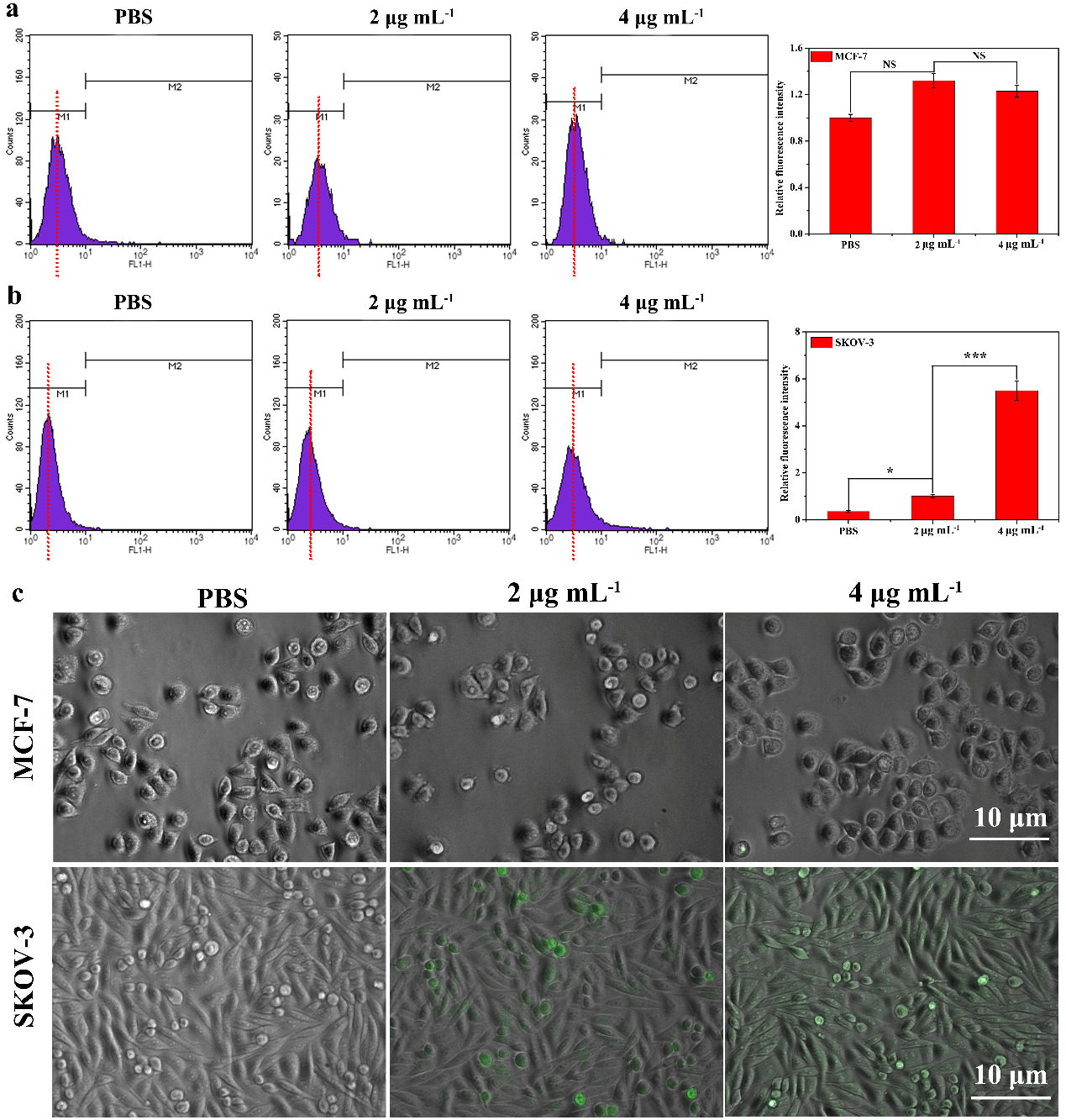


**Fig. S6.** FCM data for (a) MCF-7 or (b) SKOV-3 cells after treatment with PBS, 2 or 4 μg/mL of FITC-labelled anti-Her2 antibody for 2 h and showing the corresponding quantified fluorescence intensity. (c) Fluorescence images for MCF-7 or SKOV-3 cells after treatment with PBS and 2 or 4 μg/mL of FITC-labelled anti-Her2 antibody for 2 h.


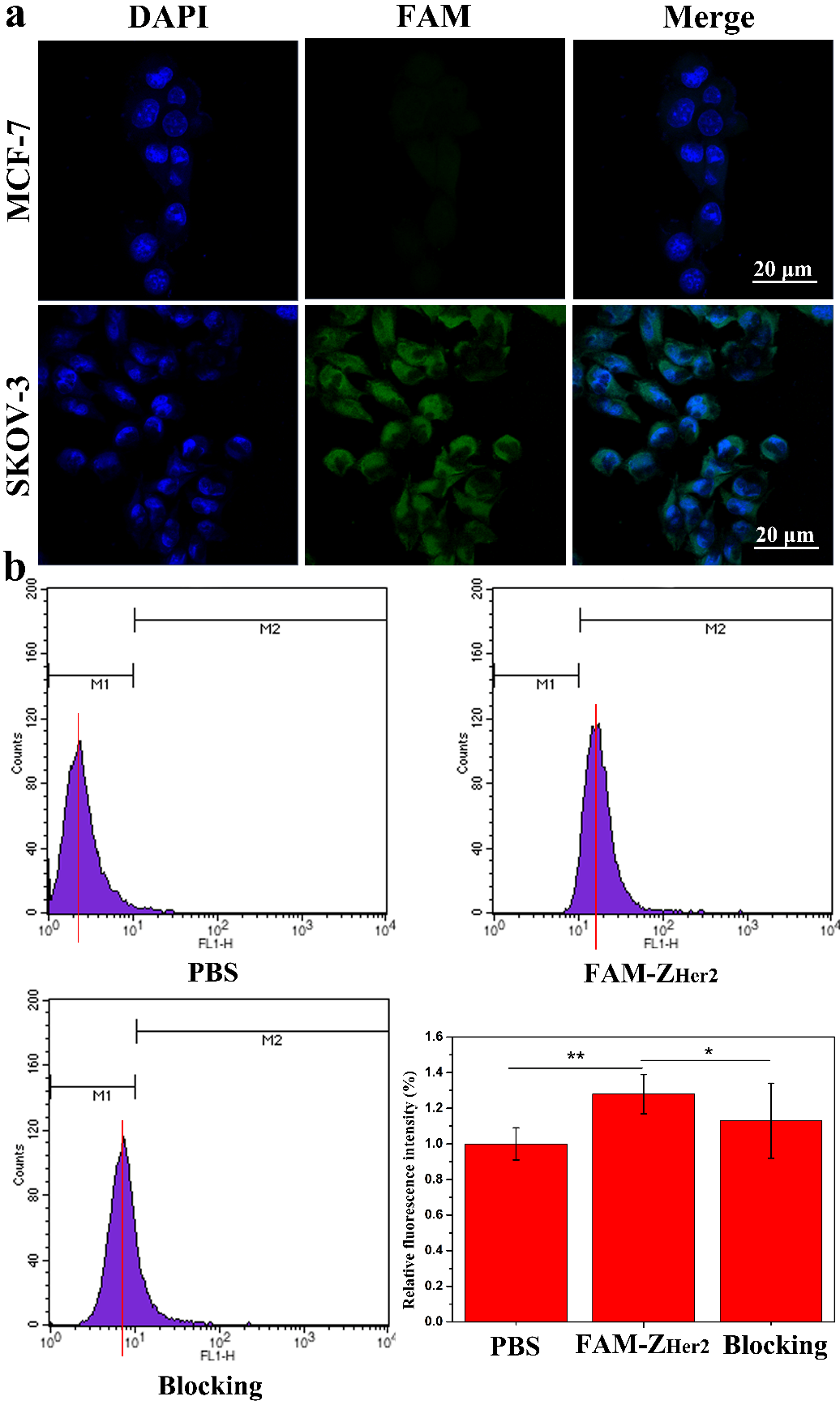


**Fig. S7.** (a) CLSM images of MCF-7 and SKOV-3 cells after 2 h incubation with FAM-Z_Her2_. (b) Flow cytometry data for: untreated SKOV-3 cells; SKOV-3 cells incubated for 2 h with FAM-Z_Her2_ and cells pre-incubated with Z_Her2_ for 1 h before being exposed to FAM-Z_Her2_ for 2 h, and corresponding quantified fluorescence intensity.


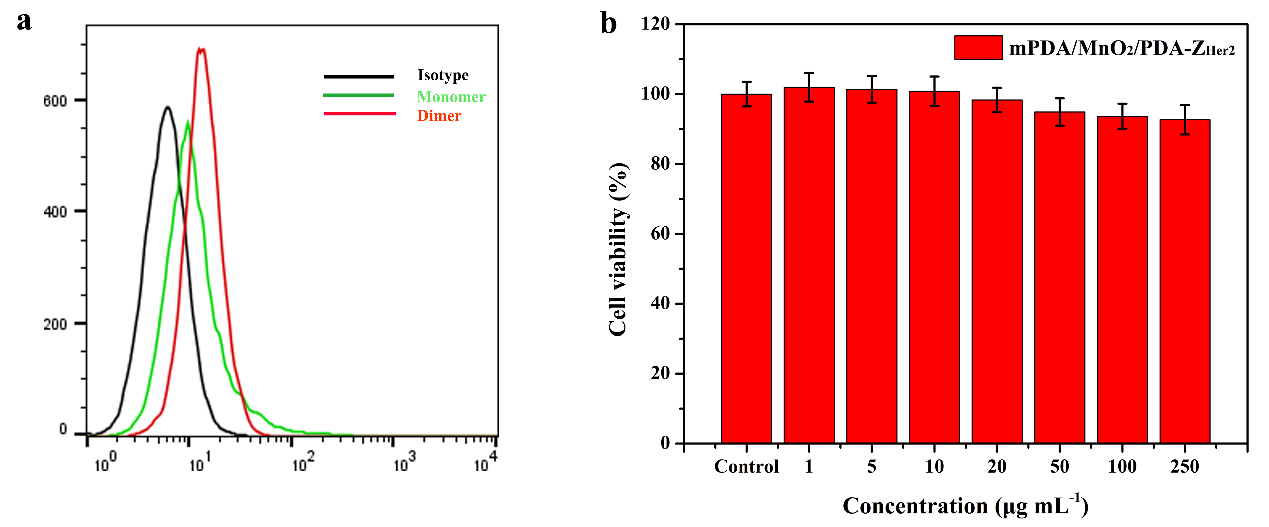


**Fig. S8.** (a) Flow cytometry data for: untreated SKOV-3 cells; SKOV-3 cells incubated for monomeric (in the presence of 2-ME) or dimeric (in the absence of 2-ME) FAM-Her2 affibody at 37 °C for 2 h. (b) MTT viability results for HUVECs treated with different concentration of mPDA/MnO_2_/PDA-Z_Her2_ NPs. Data are shown as the mean ± S.D. from three independent experiments.


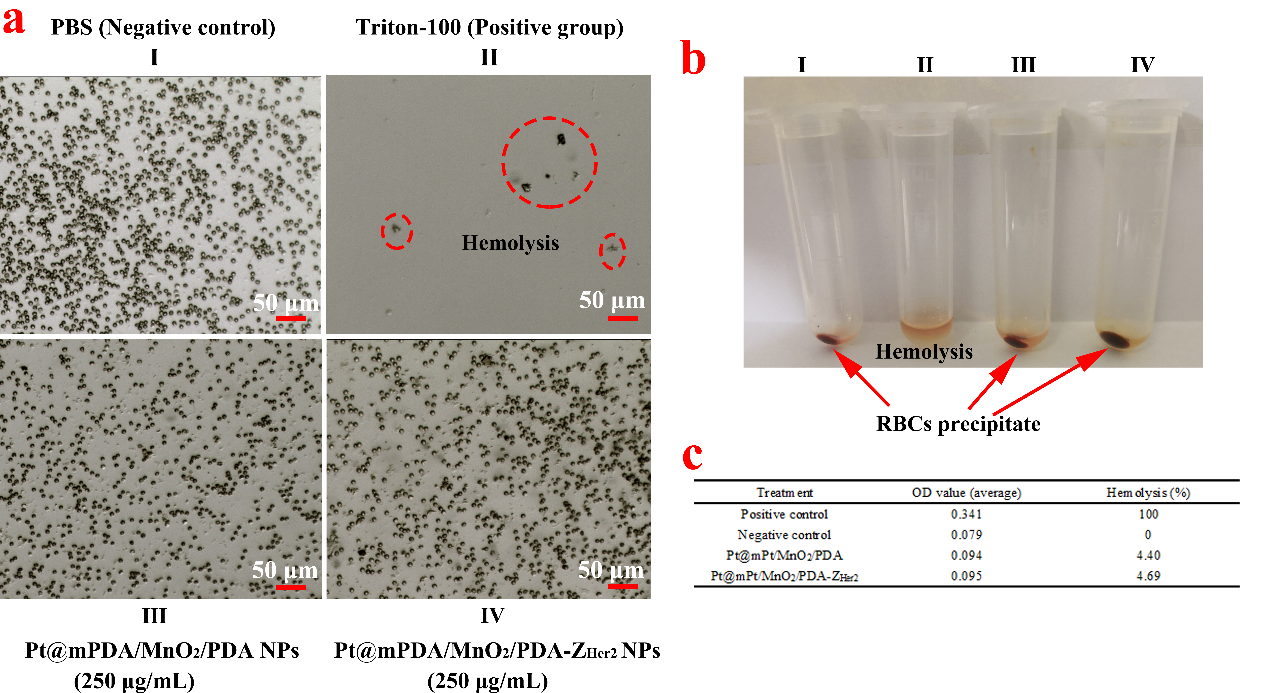


**Fig. S9.** (a) Microscope images of RBCs incubated with (Ⅰ) PBS, (Ⅱ) Triton-100, (Ⅲ) Pt@mPDA/MnO_2_/PDA NPs, (Ⅳ) Pt@mPDA/MnO_2_/PDA-Z_Her2_ NPs and (b) corresponding pictures after centrifugation (5000 rpm, 10 min). (c) Hemocompatibility data. Each number indicating the average of three times spectroscopic measurements.


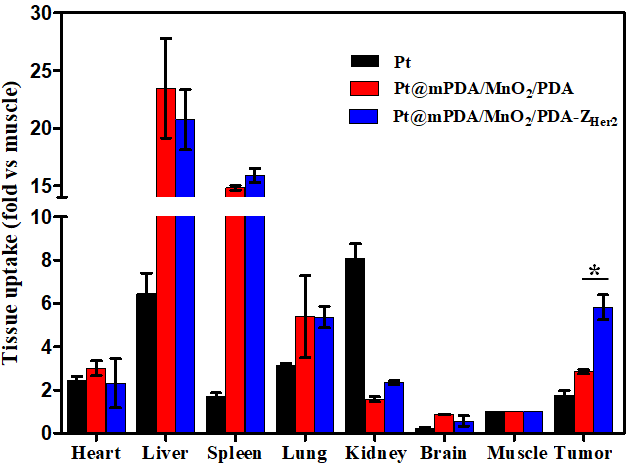


**Fig. S10.** Biodistribution of free cisplatin (Pt), Pt@mPDA/MnO_2_/PDA and Pt@mPDA/MnO_2_/PDA-Z_Her2_ NPs in mice bearing SKOV-3 tumor grafts. The tumor-bearing mice were intravenously injected with free cisplatin, Pt@mPDA/MnO_2_/PDA or Pt@mPDA/MnO_2_/PDA-Z_Her2_ NPs (dose of cisplatin = 2 mg/kg). The heart, liver, spleen, lung, kidney, brain, muscle, and tumors were extracted and weighed at 12 h after injection. Then the tissues were dissolved in aqua regia solution (2 mL; 65 °C) for 24 h and the Pt content present in different tissues were quantified by ICP-AES.


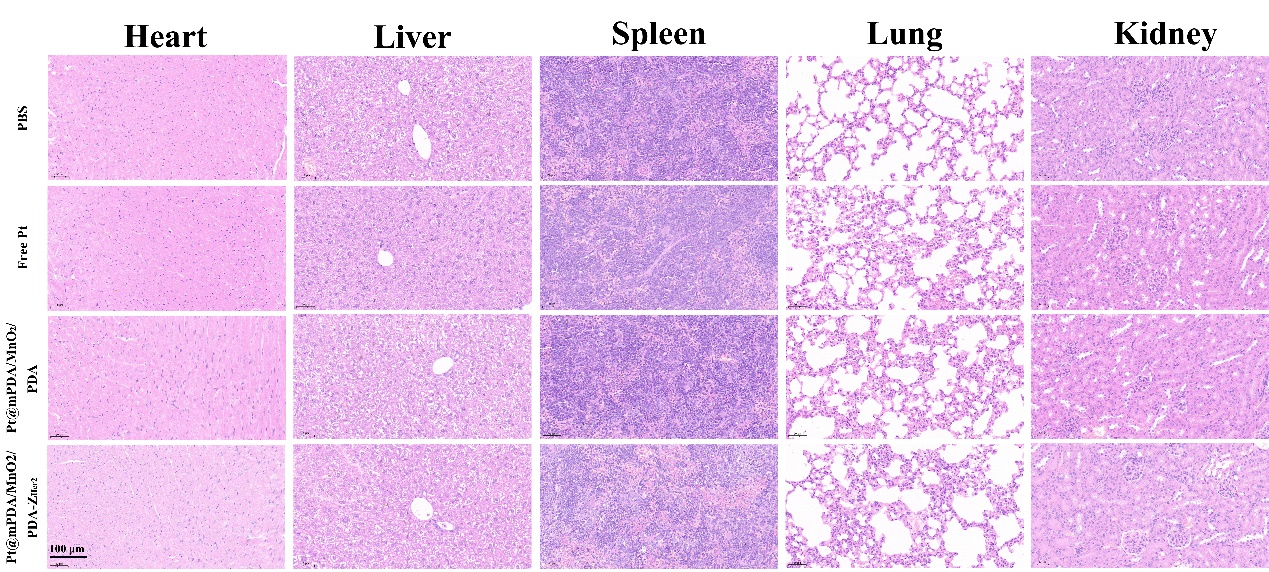


**Fig. S11.** Representative H&E-stained images of the major organs collected after *in vivo* chemotherapy experiment.

**

Fig. S12.** Blood biochemical analyses of the mice from chemotherapy experiment. 0.5 mL of blood from each group of mice (n = 3) was obtained for biochemistry analysis after the chemotherapy experiment was halted.
